# Human Gain-of-Function *MC4R* Variants Show Signaling Bias and Protect against Obesity

**DOI:** 10.1016/j.cell.2019.03.044

**Published:** 2019-04-18

**Authors:** Luca A. Lotta, Jacek Mokrosiński, Edson Mendes de Oliveira, Chen Li, Stephen J. Sharp, Jian’an Luan, Bas Brouwers, Vikram Ayinampudi, Nicholas Bowker, Nicola Kerrison, Vasileios Kaimakis, Diana Hoult, Isobel D. Stewart, Eleanor Wheeler, Felix R. Day, John R.B. Perry, Claudia Langenberg, Nicholas J. Wareham, I. Sadaf Farooqi

**Affiliations:** 1 University of Cambridge MRC Epidemiology Unit, Wellcome Trust-MRC Institute of Metabolic Science, Addenbrooke’s Hospital, Cambridge CB2 0QQ, UK; 2 University of Cambridge Metabolic Research Laboratories and NIHR Cambridge Biomedical Research Centre, Wellcome Trust-MRC Institute of Metabolic Science, Addenbrooke’s Hospital, Cambridge CB2 0QQ, UK

**Keywords:** obesity, MC4R, melanocortin, β-arrestin, UK Biobank, genetics, biased signaling, GPCRs

## Abstract

The melanocortin 4 receptor (MC4R) is a G protein-coupled receptor whose disruption causes obesity. We functionally characterized 61 *MC4R* variants identified in 0.5 million people from UK Biobank and examined their associations with body mass index (BMI) and obesity-related cardiometabolic diseases. We found that the maximal efficacy of β-arrestin recruitment to MC4R, rather than canonical Gα_s_-mediated cyclic adenosine-monophosphate production, explained 88% of the variance in the association of *MC4R* variants with BMI. While most *MC4R* variants caused loss of function, a subset caused gain of function; these variants were associated with significantly lower BMI and lower odds of obesity, type 2 diabetes, and coronary artery disease. Protective associations were driven by *MC4R* variants exhibiting signaling bias toward β-arrestin recruitment and increased mitogen-activated protein kinase pathway activation. Harnessing β-arrestin-biased MC4R signaling may represent an effective strategy for weight loss and the treatment of obesity-related cardiometabolic diseases.

## Highlights

• 61 variants in the Melanocortin-4 Receptor gene were found in 0.5 million people
• Variants causing a gain of function were associated with protection from obesity
• Variants biased toward β-arrestin signaling mediated the protective effects

## Graphical Abstract

**Figure:**
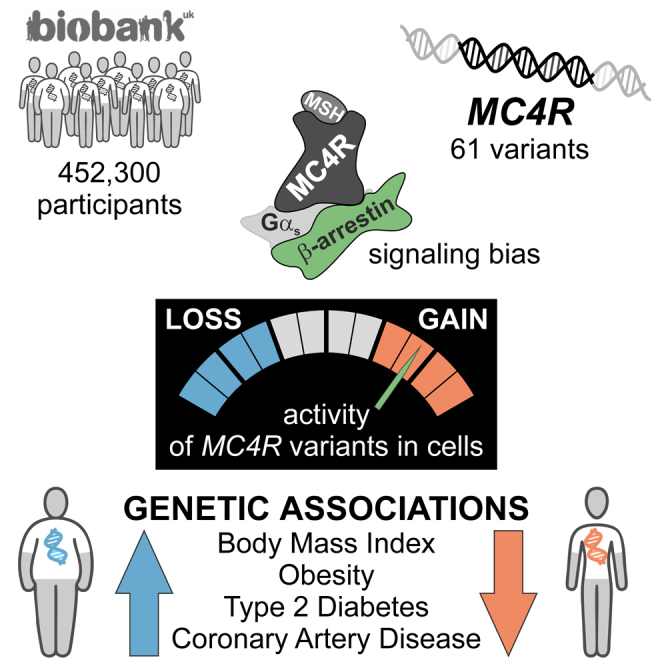

## Teaser

Gain-of-function genetic variants in the Melanocortin-4 Receptor associated with protection against obesity exhibit signaling bias for the recruitment of β-arrestin rather than canonical Gα_s_-mediated cAMP production.

## Introduction

Obesity is associated with type 2 diabetes and coronary artery disease, which together account for significant morbidity, mortality, and substantial healthcare costs globally (Heymsfield and Wadden, 2017). While advances in our understanding of the molecular mechanisms involved in weight regulation have informed the development of new weight-loss therapies, some drugs lack target specificity, while others affect multiple signaling pathways downstream of their intended target, leading to adverse effects that limit their long-term use (Bray et al., 2016). Therefore, there is a substantial unmet need for safe and effective weight-loss therapies.

G protein-coupled receptors (GPCRs) are targeted by approximately 30% of US Food and Drug Administration (FDA)-approved medicines, highlighting their tractability for drug discovery (Hauser et al., 2017, Santos et al., 2017). Classically, upon ligand binding, GPCRs interact with heterotrimeric guanine nucleotide-binding (G) proteins to direct signaling and gene transcription, a response that is attenuated within minutes when phosphorylated GPCRs bind β-arrestins, which sterically prevent their coupling to G proteins (Rajagopal and Shenoy, 2018). This molecular interaction also promotes the internalization of ligand-bound receptors to early endosomes, from where GPCRs either recycle rapidly to the cell membrane or translocate to lysosomes for degradation (Shenoy and Lefkowitz, 2011, Shinyama et al., 2003). β-arrestins may also directly/indirectly mediate signaling via mitogen-activated protein kinase (MAPK)-mediated phosphorylation of extracellular signal-regulated kinase 1/2 (ERK1/2).

While balanced GPCR agonists signal with comparable efficacy through multiple pathways, the development of biased agonists, which preferentially activate signaling through either G protein-dependent or G protein-independent β-arrestin-mediated pathways, is emerging as a powerful way of emphasizing favorable signals, while de-emphasizing signals that may lead to adverse effects (Povsic et al., 2017, Rajagopal et al., 2011, Smith et al., 2018). Such targeted drug discovery relies on the precise delineation of the relative contributions of G proteins versus β-arrestins to the physiological consequences of GPCR activation.

Here, we focused on the melanocortin 4 receptor (MC4R), a brain-expressed Gα_s_-coupled GPCR involved in weight regulation (Fan et al., 1997, Kishi et al., 2003, Mountjoy et al., 1994, Ollmann et al., 1997). Feeding-induced release of the melanocortin peptides, α- and β-melanocyte-stimulating hormone (MSH), leads to activation of MC4R-expressing neurons, resulting in reduced food intake (Cowley et al., 2001, Fan et al., 1997). Targeted deletion of *Mc4r* in rodents causes weight gain in a gene-dosage-dependent manner (Huszar et al., 1997). In humans, rare heterozygous *MC4R* variants that reduce Gα_s_-mediated cyclic adenosine monophosphate (cAMP) accumulation in cells have been identified in obese children and adults in many populations (Vaisse et al., 1998, Yeo et al., 1998) (https://www.mc4r.org.uk/). MC4R deficiency in rodents and humans (Fan et al., 2000, Farooqi et al., 2003) is characterized by low blood pressure (BP; for the degree of obesity) due to impaired sympathetic nervous system activation (Greenfield et al., 2009, Sayk et al., 2010, Simonds et al., 2014, Tallam et al., 2005). As predicted by these genetic findings, first-generation MC4R agonists caused weight loss but increased BP (Greenfield et al., 2009), which halted their development. A second-generation MC4R agonist reduced weight in rare patients with obesity due to genetic disruption of the melanocortin pathway (Clément et al., 2018, Collet et al., 2017, Kühnen et al., 2016) without affecting BP (Chen et al., 2015, Kievit et al., 2013); however, off-target effects on the melanocortin-1 receptor (skin pigmentation) may limit its wider use. We hypothesized that a more refined understanding of MC4R signaling and its impact on clinical phenotypes in the general population may inform the design of drugs targeting this pathway to treat common obesity and its complications.

We performed genetic association studies in approximately 0.5 million people from UK Biobank, focusing on 61 nonsynonymous variants identified in *MC4R*. 12 of the 61 were nonsense/frameshift variants; the remainder (n = 49) were missense variants whose functional properties were characterized in cells quantifying canonical Gα_s_-mediated cAMP production and the recruitment of β-arrestin to MC4R. In meta-regression analyses using the functional consequence of *MC4R* variants as the predictor, we found that 88% of the variance in the association of different *MC4R* variants with BMI was explained by their effect on β-arrestin recruitment. A subset of individuals (6%, n = 28,161) were carriers for gain-of-function (GoF) alleles that exhibited signaling bias, preferentially increasing β-arrestin recruitment rather than cAMP production. These individuals had significantly lower BMI (p = 2 × 10^−42^) and up to 50% lower risk of obesity, type 2 diabetes, and coronary artery disease. Cumulatively, the characterization of BMI-lowering variants in *MC4R* demonstrates the pivotal role of β-arrestin-mediated MC4R signaling in human energy homeostasis. These findings have relevance for the development of β-arrestin-biased MC4R agonists for weight loss and for the treatment of obesity-associated metabolic disease.

## Results

### Genetic Variants in *MC4R* Found in the General Population Cause Loss or Gain of Function in Cells

We studied 61 independent nonsynonymous variants in *MC4R* (pairwise R^2^ < 0.01; variant allele frequency 2%–0.0001%) that were directly genotyped (n = 59) or well imputed (info score > 0.8; n = 2) in 452,300 European ancestry participants from UK Biobank (Table S1; STAR Methods), which is a UK population-based cohort of people aged 40–69 years (Bycroft et al., 2018, Sudlow et al., 2015). Of the 61 *MC4R* variants, 12 were nonsense/frameshift variants and 49 were missense variants (Table S1).

**Figure 1:**
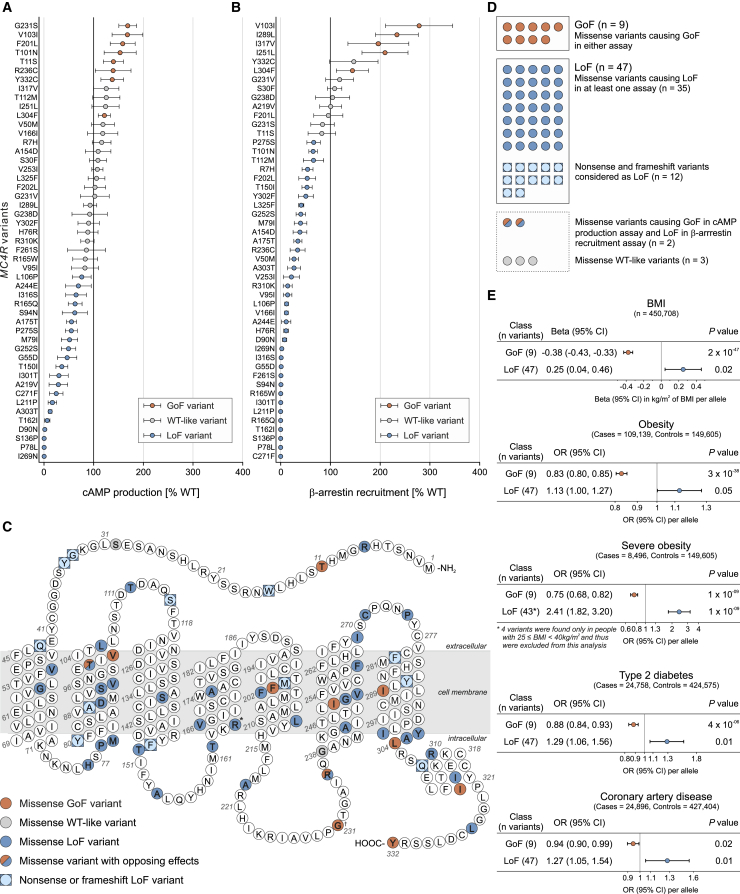
Gain-of-Function *MC4R* Variants Are Associated with Protection from Obesity and Its Complications (A and B) Maximal efficacy of (A) NDP-αMSH-induced cAMP production and (B) β-arrestin recruitment for mutant MC4Rs. Data represented as mean (95% CI) of 4–12 independent experiments; each mutant expressed as % WT. Variants classified as GoF (orange), LoF (blue), or WT-like (gray) based on statistically significant differences between WT and mutant (unpaired single-sample t test). (C) MC4R protein highlighting amino acids affected by variants. ^∗^†Residues affected by >1 variant; ^∗^R165W (LoF) and R165Q (LoF); †G231S (GoF) and G231V (WT-like). (D) Counts for GoF (n = 9) and LoF (n = 47) variants included in genetic association analyses. Five variants with opposite effects in the two assays or WT-like results were excluded. (E) Genetic associations of GoF and LoF *MC4R* variants with BMI and obesity and its complications. OR, odds ratio; CI, confidence interval; BMI, body mass index, n, number of participants. See also Tables S1, S2, S3, and S4.

To characterize the functional consequences of all missense variants in *MC4R* (Figure 1; Table S2), HEK293 cells were transiently transfected with constructs encoding wild-type (WT) or mutant MC4Rs. We measured canonical Gα_s_-mediated signaling by quantifying the maximal efficacy of ligand (NDP-αMSH)-induced cAMP production in a time-resolved assay (Figure 1A). Additionally, we quantified the interaction between WT/mutant MC4R and β-arrestin-2 using a time-resolved enzyme complementation assay (Figure 1B). We found that 58 of 61 (95%) nonsynonymous *MC4R* variants had functional consequences; 47 (77%) resulted in a loss of function (LoF), 9 (15%) resulted in a significant GoF, 2 (3%) had opposing effects on the two signaling pathways, and 3 (5%) were wild-type like in both assays (Figures 1A–1D; Table S2). In contrast to most previous studies of human *MC4R* variants, which have measured the direct/indirect accumulation of cAMP, we find that the majority of *MC4R* variants present in UK Biobank affect both cAMP production and the recruitment of β-arrestin-2 to MC4R.

### Gain-of-Function *MC4R* Variants Are Associated with Protection against Obesity and Its Cardio-metabolic Complications

We next performed genetic association studies with a series of primary phenotypes recorded in UK Biobank: BMI and obesity, hemodynamic phenotypes known to be affected by MC4R signaling (resting heart rate, systolic and diastolic BP), and risk of type 2 diabetes and coronary artery disease. We found that LoF variants in *MC4R* were associated with higher BMI and higher odds of obesity, severe obesity, type 2 diabetes, and coronary artery disease (Figure 1E). These results align with reports of LoF *MC4R* variants identified in cohorts of obese and severely obese individuals (Farooqi et al., 2003, Hinney et al., 2006, Stutzmann et al., 2008). In contrast, we found that GoF *MC4R* variants were strongly associated with lower BMI (p = 2 × 10^−47^) and lower odds of obesity (p = 3 × 10^−38^), severe obesity (p = 1 × 10^−09^), type 2 diabetes (p = 4 × 10^−06^), and coronary artery disease (p = 0.02) (Figure 1E). GoF variants, but not LoF variants, were associated with lower diastolic BP and lower resting heart rate (Table S3).

Associations with BMI were robust in sensitivity analyses that excluded ultra-rare genetic variants (variant allele frequency < 0.001%) and factored in manually curated cluster-plot quality scores (Table S4; STAR Methods). The association of LoF variants with BMI was particularly strong for variants resulting in protein truncation or complete LoF of either pathway *in vitro* (Table S4). For six overlapping nonsynonymous variants, associations with BMI were consistent with external validation data from the GIANT consortium (Locke et al., 2015, Turcot et al., 2018) (Table S4). The association of LoF alleles in *MC4R* with type 2 diabetes was validated using exome sequencing data from the T2D Knowledge Portal; odds ratio (OR) for carriers of rare LoF variants versus noncarriers, 1.59; 95% confidence interval (CI), 1.22–2.08; p = 0.0007; *P*_heterogeneity_ compared to the estimate in UK Biobank from this study = 0.21.

### β-Arrestin-Mediated MC4R Signaling Plays a Pivotal Role in Human Weight Regulation

**Figure 2:**
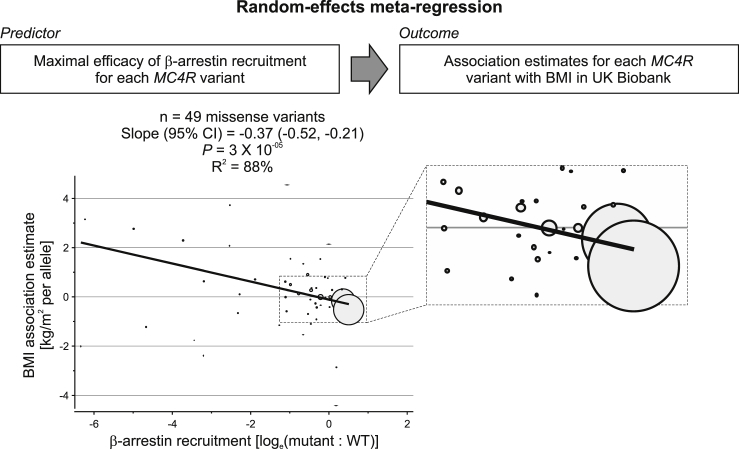
β-Arrestin Recruitment by Different *MC4R* Variants Explains a Significant Proportion of the Variance in Their Association with BMI Model (top) and results (bottom) from a meta-regression analysis in which β-arrestin recruitment for each MC4R mutant was the predictor and association estimates for BMI were the outcome. Circles represent each variant, with circle size proportional to the weight of each variant in the model. The enlarged box shows the area where variants with the largest weight clustered. CI, confidence interval; BMI, body mass index. Sensitivity analyses excluding the two variants with the largest weight and leave-one-out analyses are in Table S5.

We next used random-effects meta-regression to investigate whether β-arrestin recruitment or cAMP production explained the variance in the association of different *MC4R* variants with BMI (Figure 2; STAR Methods). We found that β-arrestin recruitment was a statistically significant predictor of the association of different *MC4R* variants with BMI (p = 3 × 10^−05^) and explained 88% of the variance in these associations (Figure 2). Several different sensitivity analyses supported the robustness of this association, including multivariable models with cAMP production as an additional predictor, leave-one-out analyses excluding one of the variants in each iteration, models restricted to rare variants (variant allele frequency < 0.5%—i.e., excluding the two variants with the largest weight in the main analysis), models including nonsense/frameshift variants, and models excluding ultra-rare genetic variants and factoring in manually curated cluster-plot quality scores (Table S5; STAR Methods). In contrast, cAMP production did not predict the associations of different *MC4R* variants with BMI either on its own (p = 0.19) or when the degree of β-arrestin recruitment was also included in the model (p = 0.52; Table S5). Increased β-arrestin recruitment also predicted lower estimates of association with BMI among the 20 variants that were WT-like for cAMP production (p = 0.02; Table S5). Increased β-arrestin recruitment remained a predictor of BMI associations when using the functional category (LoF, WT-like, or GoF) rather than the actual experimental value as predictor (p = 0.04; Table S5); there was evidence that both LoF and GoF variants contributed to this association (Table S5). Taken together, these results suggest that β-arrestin-mediated MC4R signaling plays a critical role in the regulation of human body weight.

### Gain-of-Function *MC4R* Variants that Preferentially Signal through β-Arrestin Mediate the Protective Association with BMI and Obesity and Its Complications

**Figure 3:**
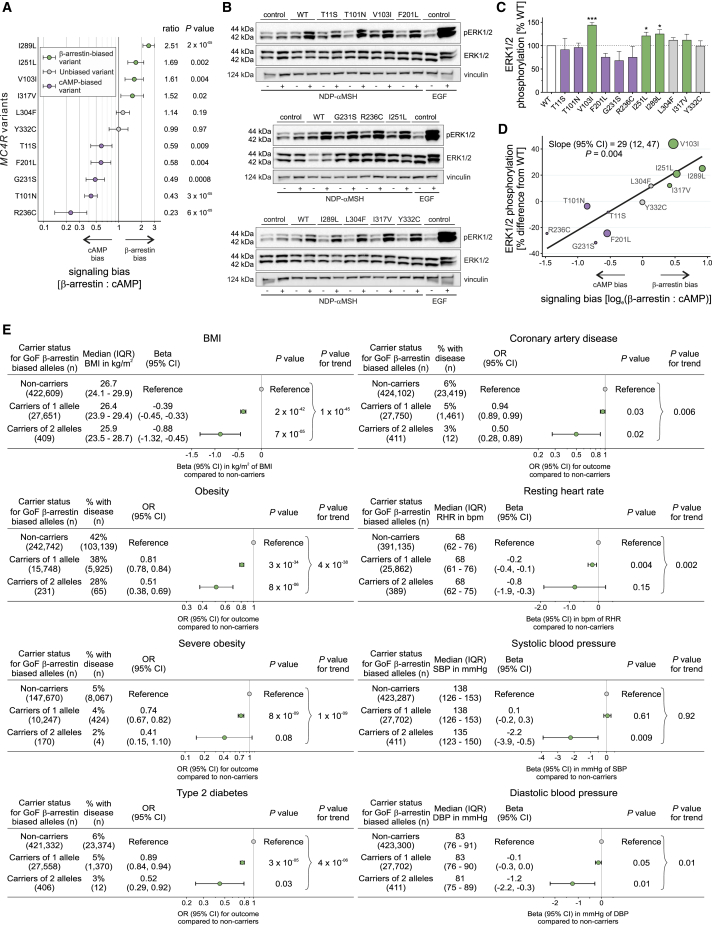
Gain-of-Function *MC4R* Variants that Exhibit Bias toward β-Arrestin-Mediated Signaling Protect against Obesity and Its Complications (A) Signaling bias for 11 GoF MC4R mutants calculated as ratio (95% CI) of geometric means for maximal activity of β-arrestin to cAMP; data from 4–12 experiments. The null hypothesis of no bias (ratio = 1) was tested using unpaired two-sample t test. Variants were classified as biased toward β-arrestin (green), biased toward cAMP (purple), or unbiased (gray). (B and C) (B) Representative western blots and (C) quantification of ERK1/2 phosphorylation (expressed as % WT) before (−) and after (+) NDP-αMSH stimulation of GoF MC4R mutants; epidermal growth factor (EGF) used as a positive control; vinculin used as a loading control. Data represented as mean ±SEM from 3–8 independent experiments; statistical significance of differences between WT and mutant (unpaired single-sample t test). (D) Meta-regression analysis showing that greater bias for β-arrestin recruitment predicts greater ERK1/2 phosphorylation for GoF variants (depicted as circles with size proportional to precision in ERK1/2 phosphorylation estimates). (E) Associations with BMI, obesity, severe obesity, type 2 diabetes, coronary artery disease, resting heart rate (RHR) in beats/min (bpm), and systolic and diastolic blood pressure (SBP and DBP, respectively) in millimeters of mercury (mmHg) by carrier status for β-arrestin-biased GoF *MC4R* alleles. OR, odds ratio; CI, confidence interval; IQR, interquartile range; n, number of participants. See also Tables S6 and S7.

We hypothesized that naturally occurring genetic variants that preferentially affect signaling through one pathway versus the other (exhibit bias) may provide insights into the physiological consequences of targeting a specific pathway therapeutically. Among 11 variants resulting in a GoF (including two that were GoF for cAMP but LoF for β-arrestin), five (T11S, T101N, F201L, G231S, R236C) exhibited significant bias toward cAMP production, four (V103I, I251L, I289L, I317V) exhibited significant bias toward β-arrestin recruitment, and two (L304F, Y332C) showed no evidence of biased signaling (Figure 3A). GoF MC4R mutants that led to increased β-arrestin recruitment (but not those that predominantly increased cAMP production) resulted in enhanced signaling via the MAPK pathway measured by quantifying ERK1/2 phosphorylation assayed using western blotting (Figures 3B–3D).

In UK Biobank, approximately 1 in every 16 participants (6.1%; n = 27,750) carried one copy of a β-arrestin-biased GoF allele, while 1 in every 1,102 (0.1%; n = 411) carried two alleles. Carriers of one GoF allele had a BMI that was on average 0.39 kg/m^2^ lower than noncarriers (p = 2 × 10^−42^; Figure 3E), while carriers of two alleles had a BMI that was 0.88 kg/m^2^ lower (p = 7x10^−05^; Figure 3E). The latter is equivalent to ∼2.5 kg lower body weight for a person 1.7 m tall. Carriers of two β-arrestin-biased GoF alleles had an approximately 50% lower risk of obesity (OR, 0.51; p = 8 × 10^−06^; Figure 3E), type 2 diabetes (OR, 0.52; p = 0.03; Figure 3E), and coronary artery disease (OR, 0.50; p = 0.02; Figure 3E) compared to noncarriers; carriers of one allele had intermediate risk (Figure 3E). Conversely, carriers of GoF variants exhibiting bias toward cAMP production had similar BMIs, and risks of obesity and cardio-metabolic disease as noncarriers; these variants were associated with a significant increase in systolic and a marginal (but nonsignificant) increase in diastolic BP compared to noncarriers (Tables S3 and S6).

Associations of β-arrestin-biased GoF alleles in *MC4R* with cardio-metabolic outcomes were directionally consistent with those observed with a 97-variant polygenic score for lower BMI derived from a previous genome-wide study (Locke et al., 2015). For coronary artery disease, the OR (95% CI) per kg/m^2^ genetically lower BMI, was 0.94 (0.93–0.95) for the 97-variant polygenic score versus 0.82 (0.72–0.95) for the β-arrestin-biased *MC4R* GoF variants, *P*_heterogeneity_ = 0.07. Interestingly, *MC4R* GoF variants were more strongly associated with a reduced risk of type 2 diabetes (OR [95% CI] per kg/m^2^ genetically lower BMI, 0.86 [0.85–0.87] for the 97-variant polygenic score versus 0.72 [0.62–0.83] for the β-arrestin-biased *MC4R* GoF variants, *P*_heterogeneity_ = 0.01). Experimental studies in rodents and humans have shown that impaired MC4R signaling increases insulin secretion (Fan et al., 2000, Greenfield et al., 2009), which may affect the onset and prevalence of type 2 diabetes in variant carriers through mechanisms that require further exploration.

### The Most Frequent Gain-of-Function *MC4R* Variant (V103I) Leads to Decreased Agonist-Induced Internalization of Mutant Receptors

**Figure S1:**
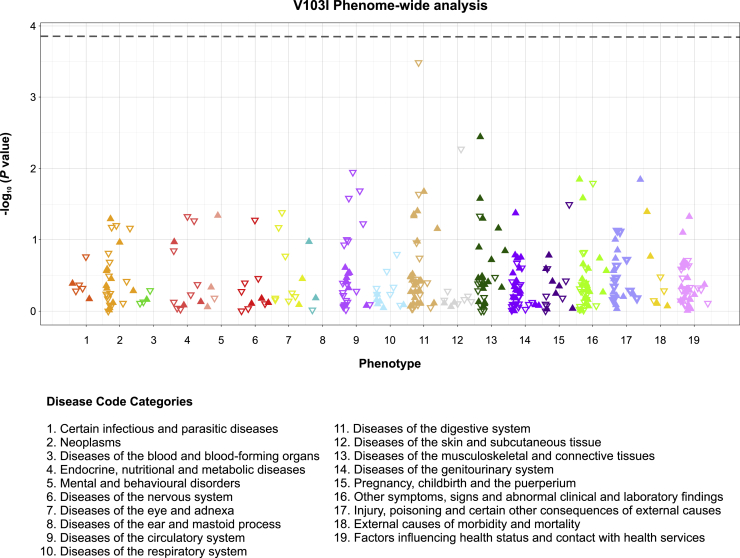
Phenome-wide Scan of the Associations of V103I *MC4R* with 353 Common Diagnoses Analyses were performed in European ancestry participants of UK Biobank. Only diagnosis codes with > 500 cases were included. Diagnosis codes were grouped in 19 broad categories. Each triangle represents the association for a given diagnosis code. Full triangles that are upward-pointing represent associations in a risk increasing direction, while open triangles that are downward-pointing represent associations in a protective direction. The horizontal broken line represents the statistical significance threshold of p < 0.00014, corresponding to a Bonferroni correction for 353 diagnoses. Related to Figure 4.

To explore the potential mechanisms by which increased β-arrestin recruitment leads to a GoF rather than to a LoF as might be predicted, we studied V103I MC4R, the commonest nonsynonymous variant (variant allele frequency, 2%) found in UK Biobank which exhibits significant bias toward β-arrestin-mediated signaling (Figure 3A; p = 0.004). Previously, we and others have reported associations of V103I *MC4R* with lower BMI and obesity risk (Geller et al., 2004, Gu et al., 1999, Heid et al., 2005, Stutzmann et al., 2007, Young et al., 2007), which were confirmed in this analysis (Table S7). In meta-analyses of genetic association studies including over 600,000 people, we now find that V103I *MC4R* is associated with lower risk of type 2 diabetes (p = 7 × 10^−07^) and of coronary artery disease (p = 0.003; Table S7). V103I *MC4R* was also associated with lower diastolic BP and resting heart rate, but not with any adverse disease outcomes in an exploratory phenome-wide association analysis of 353 frequent clinical diagnoses in UK Biobank (Figure S1).

**Figure 4:**
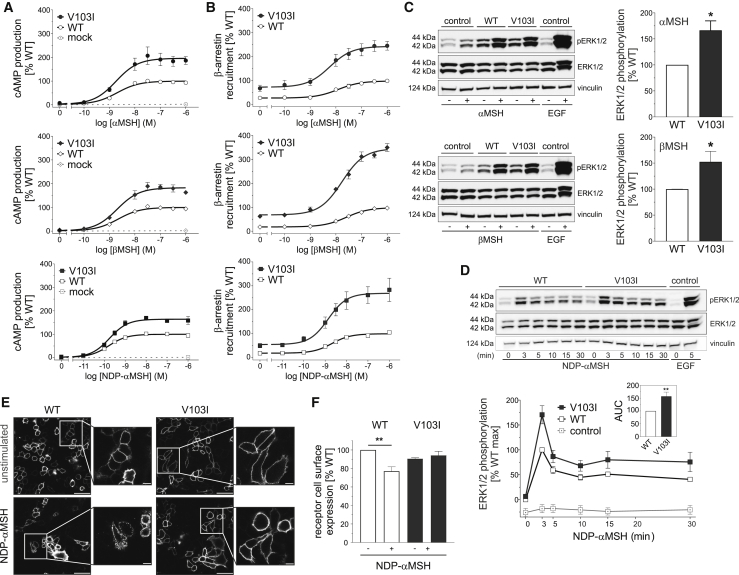
Effects of V103I MC4R on Signaling and Receptor Internalization (A and B) Dose-response curves for αMSH-, βMSH-, and NDP-αMSH-induced (A) cAMP production and (B) β-arrestin recruitment for V103I compared to WT MC4R and mock transfected cells expressed as % WT. Data represented as mean ± SEM from 4 independent experiments. (C and D) Representative western blots and quantification of ERK1/2 phosphorylation by WT and V103I MC4R before (−) and after (+) stimulation by (C) αMSH and βMSH and (D) NDP-αMSH in a time-course experiment; EGF used as a positive control; vinculin used as a loading control. (E and F) (E) Confocal microscopy and (F) receptor cell surface expression quantified by FACS for cells expressing WT and V103I MC4R before (−) and after (+) stimulation by NDP-αMSH. Scale bars, 50 μm; 10 μm (inset). Data represented as mean ± SEM from 3–8 independent experiments; statistical significance of differences between WT and mutant analyzed with unpaired single-sample t test (A, B, C, D, F). ^∗^p < 0.05; ^∗∗^p < 0.01. AUC; area under the curve. See also Figures S1 and S2 and Table S7 and S8.

**Figure S2:**
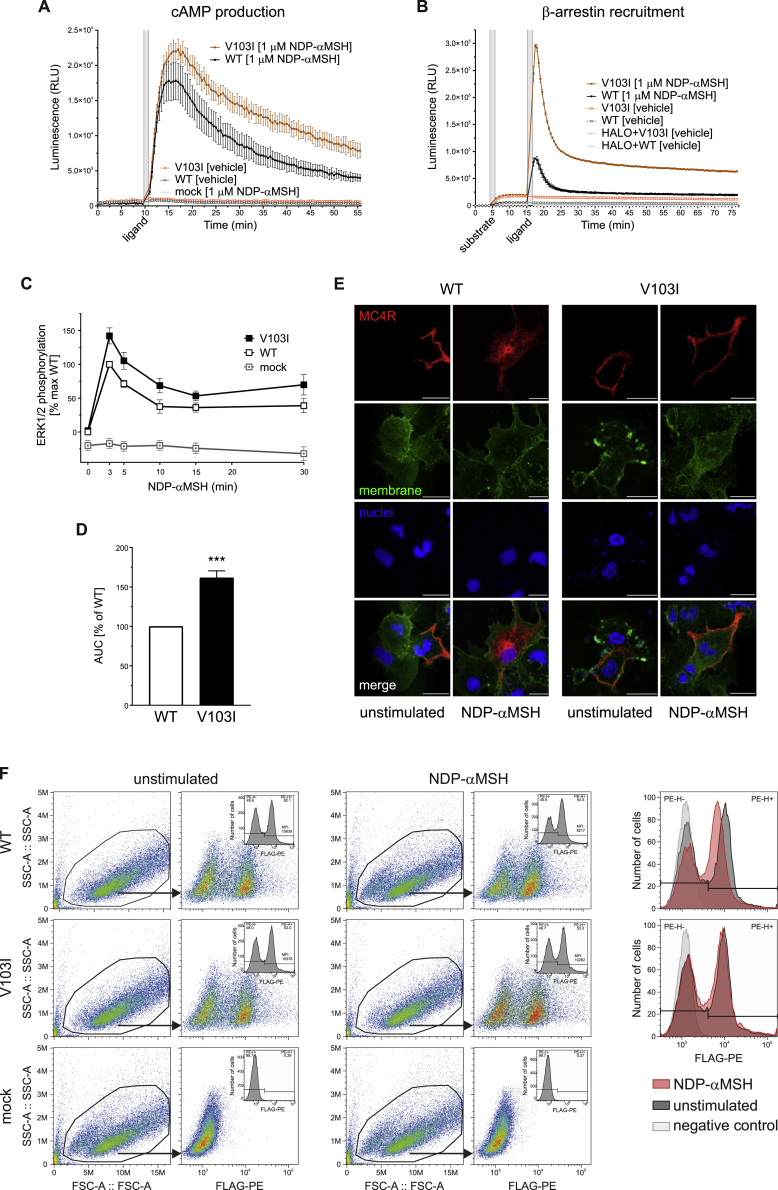
Effects of V103I MC4R on cAMP Production, β-arrestin Recruitment, MAPK Pathway Activation, and Cell Surface Expression Representative real-time measurement of (A) cAMP (B) β-arrestin recruitment upon NDP-αMSH stimulation. (C) Time-course quantification and (D) respective area under the curve (AUC) of ERK1/2 phosphorylation assessed by Homogeneous Time-Resolved Resonance Energy Transfer (HTRF)-based sandwich immunoassay (Cisbio, 64AERPET); data expressed as % WT. WT (open squares), V103I (solid squares) MC4R; mean ± standard error; (n = 4); statistical significance of differences between WT and mutant analyzed in an unpaired single-sample t test; ^∗∗^p < 0.01. (E) Confocal microscopy on COS-7 cells expressing WT and V103I MC4R unstimulated and upon NDP-αMSH stimulation. MC4R in red (Anti-FLAG (M2) antibody), plasma membrane in green (wheat germ agglutinin) and nuclei in blue (DAPI). Scale bars, 50 μm. (F) Effects of V103I MC4R on receptor internalization quantified by FACS. Representative data on receptor internalization using HeLa cells transiently transfected with FLAG-MC4R WT or V103I. Mock-transfected cells were used as controls for non-specific binding of the antibody (FLAG-PE; negative control). Mean fluorescence intensity (MFI) represents the amount of MC4R present at the cell surface; receptor internalization was quantified using the mean fluorescence intensity (MFI) values of FLAG-PE^+^ cells from unstimulated (-) and NDP-αMSH stimulated WT and V103I MC4R. Related to Figure 4.

**Figure 5:**
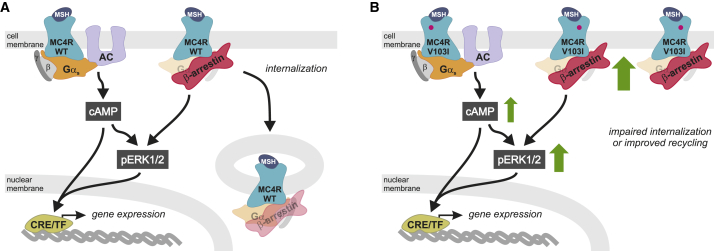
Mechanisms by Which V103I MC4R Causes a Gain-of-Function (A and B) Schematic of mechanisms by which V103I MC4R (pink dot) results in GoF. G proteins (α,β,γ); MSH, melanocyte stimulating hormone; AC, adenylate cyclase; CRE, cyclic AMP response element; TF, transcription factor.

In contrast to previous studies (Gu et al., 1999, Hinney et al., 2006), we found that V103I MC4R increased ligand-induced cAMP production in a time-resolved assay (Figures 4A and S2A; Table S8). β-arrestin recruitment by V103I MC4R was significantly increased in response to both synthetic and endogenous ligands, an effect that was sustained over 60 min (Figures 4B and S2B; Table S8). The magnitude and duration of ligand-induced ERK1/2 phosphorylation was also increased (p = 0.009) (Figures 4C–4D, S2C, and S2D). Using confocal microscopy, we demonstrated that while WT MC4Rs translocated from the membrane into the cytoplasm upon agonist stimulation, V103I MC4R remained at the cell surface (Figures 4E and S2E). These findings were replicated in a fluorescence-activated cell sorting (FACS) assay where cell surface expression of WT MC4R decreased by 23% upon ligand stimulation (p = 0.003; Figure 4F), while there was no change in expression of V103I MC4R (Figure 4F and S2F). Further studies will be needed to investigate whether these findings may be explained by impaired internalization or accelerated recycling of V103I MC4Rs, leading to an accumulation of V103I MC4R at the cell surface and a GoF (Figure 5).

## Discussion

By combining genetic studies in over 0.5 million people with detailed functional characterization of identified *MC4R* variants in cells, we demonstrated that β-arrestin-biased GoF *MC4R* variants are associated with lower risk of obesity and its cardio-metabolic complications in the general population. We found that almost all naturally occurring nonsynonymous variants in *MC4R* affect signaling and that the degree of β-arrestin recruitment to MC4R accounts for a large proportion of the variation in genetic association of these *MC4R* variants with BMI in the general population, indicating that MC4R signaling through β-arrestin is critical for its role in the regulation of body weight. Approximately 6% of European-ancestry individuals in the general United Kingdom population carry β-arrestin-biased GoF variants, which are associated with up to 50% lower risk of obesity and its metabolic complications but are not associated with increased BP and heart rate (HR). These findings provide strong human genetic evidence to inform the development of β-arrestin-biased MC4R agonists for weight loss and the treatment of obesity-associated metabolic disease.

### The Discovery of Protective Human Genetic Variants

It is well-established that human genetic studies can inform the understanding of disease mechanisms and the development of new therapeutics. This concept is illustrated by the rapid development of new lipid-lowering drugs guided by studies of LoF and GoF coding variants in *PCSK9* (Cohen et al., 2006), *LPA* (Clarke et al., 2009), *APOC3* (Crosby et al., 2014, Jørgensen et al., 2014), and *ANGPTL3* (Dewey et al., 2017, Musunuru et al., 2010) and by the higher probability of successful drug development for targets supported by human genetic evidence (Nelson et al., 2015, Plenge et al., 2013).

In addition to studies of genetic variants that cause or are associated with disease or risk of disease, an alternative approach gaining traction in several fields is the study of “resilient” individuals (e.g., smokers who remain healthy) or extremely elderly and healthy individuals (centenarians) (Friend and Schadt, 2014, Govindaraju et al., 2015, Harper et al., 2015). Several protective alleles have been identified to date (Liu et al., 1996, Stitziel et al., 2014). Some of these are rare and ancestry specific, for example, a LoF allele within the amyloid-β precursor protein (*APP*)-coding region in Icelanders reduces amyloid-β aggregation and may offer protection against Alzheimer’s disease (Goate, 2006). Scandinavian carriers of variants in SLC30A8 (Solute carrier family 30, member 8) are significantly less likely to develop type 2 diabetes even if obese (Flannick et al., 2014)—associations that have been replicated in people from other ancestries. However, the discovery of low-frequency variants associated with protection from common complex diseases is contingent upon sample size, with large numbers of affected individuals and controls being required to generate sufficient power to detect these associations. Here, by studying data on BMI and metabolic diseases in 0.5 million participants in UK Biobank and by focusing on a gene known to be involved in the regulation of weight and harboring a large number of low-frequency variants, we find BMI-lowering genetic variants that are prevalent in a significant proportion (6%) of European ancestry individuals.

Our study has demonstrated the value of testing the functional consequences of variants identified in large-scale genetic association studies, in particular as GoF variants cannot be reliably identified or predicted using *in silico* algorithms. Here, by measuring the functional consequences of all missense *MC4R* variants in cells, we demonstrate strong associations for GoF variants with lower risk of obesity and show that LoF variants are associated with obesity and diabetes risk in the general population. LoF *MC4R* variants were first identified in people with hyperphagia and severe early-onset obesity 20 years ago (Vaisse et al., 1998, Yeo et al., 1998), and subsequently, over 300 rare variants that reduce cAMP accumulation have been identified, mostly in obese people (Collet et al., 2017, Stutzmann et al., 2008) (https://www.mc4r.org.uk/). A recent study in the general population identified an association of the known Y35X/D37V haplotype with higher BMI (Turcot et al., 2018). However, this and other studies have not detected significant associations for other *MC4R* LoF variants with obesity in the general population (Hinney et al., 2006). We suggest that these discordant findings may be partly explained by the rarity of these variants but also by the fact that some rare variants, including several predicted damaging by *in silico* algorithms, have minimal impact on cAMP signaling but do, as shown in this study, impact on β-arrestin recruitment, which has previously not been studied. Our study highlights the value of combining detailed and comprehensive functional characterization of variants with large-scale genetic analyses.

While population-based studies may tend to underestimate the phenotypic consequences of genetic variants, as participants tend to be healthier than individuals in the general population from which they are sourced, studies of severe clinical cases may overestimate them (Wright et al., 2019). This may partly explain the smaller impact on BMI for *MC4R* LoF variants in population-based cohorts as opposed to cohorts of severely obese people (Farooqi et al., 2003). Furthermore, for a relatively modest difference in BMI (0.4 to 0.9 kg/m^2^), we observed that β-arrestin-biased GoF alleles in *MC4R* were associated with a large difference in risk of cardio-metabolic disease outcomes (up to 50% lower risk), more than expected from observational epidemiology studies (Wormser et al., 2011). This is likely to reflect the life-long nature of exposure to lower levels of the risk factor (BMI) due to genotype as opposed to short-term exposure in observational studies or clinical trials. Typical examples of this phenomenon are genetic variants associated with small differences in low-density-lipoprotein cholesterol that are associated with a large reduction in cardiovascular risk (Cohen et al., 2006, Ference et al., 2015).

### Insights into Biased Signaling from Human Variants in GPCRs

With advances in GPCR biology and in our understanding of structure-activity relationships (Whalen et al., 2011), the potential to develop biased agonists that differentially activate signaling pathways is beginning to be realized. Experiments demonstrating that morphine has greater analgesic properties and causes less respiratory depression and constipation in β-arrestin-2 knockout mice have paved the way for trials of a small-molecule μ-opioid receptor agonist that stimulates nearly undetectable levels of β-arrestin recruitment compared to morphine (Wadman, 2017). However, cell-type-specific effects on the differential propagation of signaling responses can affect the interpretation of pharmacological studies, resulting in a need to establish which signaling pathway leads to the desired therapeutic effect *in vivo* (Gundry et al., 2017). By demonstrating that GoF β-arrestin-biased *MC4R* alleles in the population are associated with up to a 50% lower risk of obesity and type 2 diabetes, our studies demonstrate that naturally occurring genetic variants in a GPCR can be used to characterize the physiological consequences of biased signaling in humans. This approach is likely to have broader relevance. By analyzing data from over 68,000 individuals, Hauser et al. have recently shown that there is substantial variation in genes encoding 108 GPCRs that are targeted by known drugs (Hauser et al., 2018). Combining genetic predictions with experiments in cells, they showed that specific variants in the μ-opioid and cholecystokinin-A receptors could affect therapeutic responses *in vitro*, which they hypothesized might predict clinical response *in vivo*. Our data suggest that the phenotypic consequences of genetic variants that exhibit natural signaling bias for a given pathway may serve as a “blueprint” for the likely consequences of preferentially modulating that pathway pharmacologically with a biased agonist. This approach may be generalizable to other GPCRs and thus to the development of a broad spectrum of drug targets.

In summary, our work has shown that dissecting the molecular mechanisms underpinning genetic associations with disease, and with protection from disease, can advance our understanding of how to most effectively target specific GPCRs for the treatment of common complex diseases such as obesity and its cardio-metabolic complications.

## STAR★Methods

### Key Resources Table

**Table:** 

REAGENT or RESOURCE	SOURCE	IDENTIFIER
**Antibodies**		

Rabbit anti-p44/42 MAPK (Erk1/2) (137F5)	Cell Signaling Technology	Cat#4695; RRID: AB_390779
Rabbit anti-Phospho-p44/42 MAPK (Erk1/2) (Thr202/Tyr204)	Cell Signaling Technology	Cat#9101; RRID: AB_331646
Rabbit-Anti-Vinculin [EPR8185]	Abcam	Cat#ab129002; RRID: AB_11144129
Goat anti-rabbit IgG-HRP	Dako	Cat#P0448; RRID: AB_2617138
Mouse monoclonal anti-FLAG (M2) antibody	Sigma-Aldrich	Cat#F1804; RRID: AB_262044
Goat anti-mouse IgG-HRP	Dako	Cat#P0447; RRID: AB_2617137
Phycoerythrin (PE)-conjugated anti-DYKDDDDK tag antibody, clone L5	Biolegend	Cat#637310; RRID: AB_2563148

**Bacterial and Virus Strains**		

XL10-Gold	Agilent	Cat# 200315

**Chemicals, Peptides, and Recombinant Proteins**		

NDP-αMSH	Bachem	Cat#H-1100
α-MSH	Bachem	Cat#H-1075
β-MSH	Bachem	Cat#H-1475
Forskolin	Sigma-Aldrich	Cat#F6886
Recombinant human epidermal growth factor (EGF)	Invitrogen	Cat#PHG0311
Lipofectamine 2000™	GIBCO	Cat#11668

**Critical Commercial Assays**		

GloSensor cAMP biosensor - pGloSensor 20F plasmid	Promega	Cat#E1171
GloSensor cAMP Reagent	Promega	Cat#E1290
NanoBiT protein:protein interaction assay	Promega	Cat#M2014
Nano-Glo Live Cell Assay System	Promega	Cat#M2013
QuickChange II XL kit	Agilent Technologies	Cat#200516

**Experimental Models: Cell Lines**		

HEK293	ATCC	CRL-1573; RRID: CVCL_0045
HeLa	Provided by Kevin Moreau (University of Cambridge)	NA

**Recombinant DNA**		

Human N-FLAG-MC4R-WT in pcDNA3.1(+) vector	Farooqi et al., 2000	NA
Human N-FLAG-MC4R variants in pcDNA3.1(+) vector	This paper	NA

**Software and Algorithms**		

STATA v14.2	StataCorp	https://www.stata.com/stata14/
R v3.2.2	The R Foundation for Statistical Computing	https://cran.r-project.org/bin/windows/base/old/3.2.2/
BOLT-LMM v2.3.2	Loh et al., 2015	https://data.broadinstitute.org/alkesgroup/BOLT-LMM/
FIJI	Schindelin et al., 2012	https://fiji.sc
FlowJo	Tree Star	https://www.flowjo.com
Prism 7	Graph Pad Software	https://www.graphpad.com/scientific-software/prism/

### Contact for Reagent and Resource Sharing

Further information and requests for resources and reagents should be directed to and will be fulfilled by the Lead Contact, I. Sadaf Farooqi (isf20@cam.ac.uk).

### Experimental Model and Subject Details

#### Studies in humans

UK Biobank is a prospective population-based cohort study of people aged 40-69 years who were recruited in 2006-2010 from 22 centers located in urban and rural areas across the United Kingdom (Sudlow et al., 2015). Participants’ characteristics are reported in Table S1. UK Biobank has received ethical approval from the North West Multicenter Research Ethics Committee and participants gave written informed consent.

#### Studies in cellular models

HEK293 (XX female) and suspension HeLa (XX female) cells were cultured in high glucose Dulbecco’s modified eagle medium (DMEM, GIBCO, 41965) and supplemented with 10% fetal bovine serum (GIBCO, 10270, South America origin), 1% GlutaMAX (100X) (GIBCO, 35050), and 100 units/mL penicillin and 100 μg/mL streptomycin (Sigma-Aldrich, P0781). Cells were incubated at 37°C in humidified air containing 5% CO_2_ and transfections were performed using Lipofectamine 2000 (GIBCO, 11668) in serum-free Opti-MEM I medium (GIBCO, 31985) according to the manufacturer’s protocols.

### Method Details

#### Studies in Humans

##### Genotype data

We studied 61 nonsynonymous genetic variants in *MC4R* (GenBank: NM_005912) that were directly genotyped or well-imputed in UK Biobank (Table S1). All participants of UK Biobank with suitable DNA samples underwent genome-wide SNP-array genotyping using the Affymetrix UK BILEVE and UK Biobank Axiom arrays, with imputation to the Haplotype Reference Consortium r1.1 panel supplemented with the 1000 Genomes phase 3 and UK10K panels, as previously described. (Bycroft et al., 2018). A total of 59 variants were directly genotyped, while 2 genetic variants were imputed and had an imputation quality score greater than 0.8, indicating high-quality imputation. The 61 variants included in this study had pairwise R^2^<0.01, consistent with no or negligible linkage disequilibrium.

##### Genotype quality checks

Genotype quality control in UK Biobank followed guidelines that have been published in detail elsewhere (Bycroft et al., 2018). In brief, DNA samples were assigned to genotype batch using an automated sample selection algorithm to ensure random assignment relative to baseline characteristics. Genotyping underwent a number of quality control procedures including (a) routine quality checks carried out during the process of sample retrieval, DNA extraction, and genotype calling; (b) checks and filters for genotype batch effects, plate effects, departures from Hardy-Weinberg equilibrium, sex effects, array effects, and discordance across control replicates; (c) individual and genetic variant call rate filters. Wright et al. (Wright et al., 2019) have proposed that the expert manual review of genotype cluster plots may help distinguish lower versus higher quality genotyped variants in UK Biobank, particularly for rare alleles. We adopted a similar scoring approach. In the aforementioned study, cluster plots for each genotyping-batch were merged into one single cluster plot. In this study, instead, we reviewed cluster-plots by genotyping-batch, which reflects the data units parsed by the genotyping algorithm and is less likely to be influenced by batch effects or variation in fluorescence signal. Cluster plots were generated using evoker-lite (https://github.com/dlrice/evoker-lite) using the default configuration for UK Biobank data. This plots the clusters in the groupings and on the axes that are used by the clustering algorithm. Each variant is plotted for each genotyping batch separately, using x axis (contrast between signals A and B) = log_2_(A/B), and y axis (signal strength) = log_2_(A^∗^B)/2. Two independent expert laboratory team members reviewed the cluster plots of each batch for each of the rare variants in *MC4R* included in the study. Blind to each other and to the association results, they scored the cluster-plot quality of each variant as low (score = 0, most cluster plots display low-quality, defined for instance by carriers being called at the edge of a the cluster of non-carriers without contrast separation or with very low signal strength), intermediate (score = 1, the majority of cluster plots display high quality, defined by separation of clusters and signal strength of carriers close to average) or high (score = 2, all or almost all cluster plots display high-quality). Individual scoring was highly consistent with ∼80% of variants receiving the same exact score and only 1 variant receiving a high-score by one scorer and a low-score by the second scorer (resolved with scoring by a third independent scorer). The results of the individual scoring were summed into an overall cluster-plot quality score and variants defined as low-quality cluster plot score if the combined score was 0 or 1, intermediate-quality if the combined score was 2, high-quality if the combined score was 3 or 4. One variant had a low-quality cluster-plot score (V166I), while two had intermediate-quality scores (G55D and F202L) and all other variants had high quality cluster-plot scores.

##### Genetic association analysis

Association of genotypes with outcome phenotypes were estimated using linear or logistic regression models, as appropriate for outcome type and analytical design. To minimize genetic confounding, association analyses were restricted to European ancestry individuals, identified by combining k-means clustering of genetic principal components with self-reported ancestry. To control for relatedness, analyses were either clustered using family structure data (third degree relatives) and adjusted for 40 genetic principal components or performed using linear mixed-effects models adjusting for a genomic kinship matrix. All analyses were adjusted for age, sex and genotyping array.

In analyses of the association of GoF or LoF variants, association estimates for each variant of either functional category were pooled using fixed-effect inverse-variance weighted meta-analysis (Burgess et al., 2013). In these analyses, GoF variants were variants with significantly enhanced cAMP production or β-arrestin recruitment compared to wild-type MC4R in experiments. LoF variants were variants with significantly reduced cAMP production or β-arrestin recruitment compared to wild-type MC4R or variants resulting in premature receptor truncation (frameshift or nonsense variants). Variants that were WT-like or had opposite effects on the two pathways (GoF for cAMP production but LoF for β-arrestin recruitment) were excluded from these analyses. Genetic association analyses were performed using STATA v14.2 (StataCorp, College Station, Texas 77845 USA), R v3.2.2 (The R Foundation for Statistical Computing), BOLT-LMM v2.3.2 (Loh et al., 2015).

##### Phenotype definitions

Primary outcomes of interest were BMI, obesity, type 2 diabetes and coronary artery disease. We also investigated associations with hemodynamic phenotypes known to be affected by MC4R signaling (Greenfield et al., 2009), i.e., resting heart rate, systolic and diastolic blood pressure. BMI was calculated as weight in kilograms divided by height in meters squared. Height was measured using a Seca 240cm tape, while weight was measured using a Tanita BC418MA body composition analyzer. Systolic, diastolic blood pressure and resting heart rate were measured at baseline using an Omron blood pressure monitor and following a standardized procedure (http://biobank.ctsu.ox.ac.uk/crystal/docs/Bloodpressure.pdf). Type 2 diabetes was defined on the basis of self-reported physician diagnosis at nurse interview or digital questionnaire, age at diagnosis older than 36 years (to exclude likely type 1 diabetes cases), use of oral anti-diabetic medications or electronic records of hospital admissions or death reporting type 2 diabetes as diagnosis or cause of death (International Statistical Classification of Diseases and Related Health Problems Tenth Revision [ICD-10] code E11). Coronary artery disease was defined as either (a) myocardial infarction or coronary disease documented in the participant’s medical history at the time of enrolment by a trained nurse or (b) hospitalization or death involving acute myocardial infarction or its complications (i.e., ICD-10 codes I21, I22 or I23). Obesity was defined on the basis of BMI greater than or equal to 30 kg/m^2^ and severe obesity as BMI greater than or equal to 40 kg/m^2^. In obesity association analyses, the control group was the group of people with BMI less than 25 kg/m^2^.

##### Meta-regression

The potential for *in vitro* measures of β-arrestin recruitment or cAMP production to explain the variance (i.e., between-genetic-variants variance) in the association of different *MC4R* genetic variants with BMI was investigated using random-effects meta-regression. In these models, the predictors were the relative E_max_ for β-arrestin recruitment and/or cAMP signaling of a given *MC4R* variant allele compared to wild-type (on a natural log-scale) measured *in vitro* as described below. Values of the outcome were the associations of each genetic variant with BMI (in kg/m^2^ per copy of variant allele), estimated in 450,708 European ancestry participants in UK Biobank using linear mixed models adjusted for age, sex and a genomic kinship matrix. For significant predictors, the percentage of total variance in the outcome explained by a given predictor (e.g., *in vitro* β-arrestin recruitment) was calculated.

Similar meta-regression analyses were conducted (1) in the overall set of 49 missense variants using the functional category of β-arrestin recruitment (ie. LoF, WT-like or GoF) to assess whether the different functional categories of genetic variants predicted their association with BMI; (2) in a subset of 20 missense variants that were found to be wild-type-like for cAMP signaling to assess whether *in vitro* β-arrestin recruitment predicted their association with BMI; and (3) in a subset of 11 gain-of-function variants to assess whether bias toward β-arrestin recruitment *in vitro* predicted their level of signaling via the ERK/MAPK pathway.

##### Sensitivity and external validation analyses

Associations with BMI of functional variants in *MC4R*: To assess whether genotyping cluster-plot quality was influencing associations with BMI, we conducted sensitivity analyses after (a) exclusion of ultra-rare genetic variants (variant allele frequency < 0.001%; ie. variants that were shown to have generally lower quality cluster-plot scores by Wright et al. (Wright et al., 2019); (b) exclusion of variants from point (a) plus any variant with low overall cluster-plot quality score; (c) exclusion of variants from points (a-b) plus any variant with intermediate cluster-plot quality score; (d) exclusion of variants from points (a-c) plus any variant where the combined cluster-plot quality score was below 4 (ie. the maximum possible score).

Meta-regression analyses: The main analysis (Figure 2) included all 49 missense variants in *MC4R* found in European ancestry participants of UK Biobank. Over 50 sensitivity analyses were conducted to assess the robustness of the results of the main analysis (Table S5). First, we conducted 49 leave-one-out analyses where each missense variant was excluded at a given iteration to assess if a single variant was driving the association observed in the main analysis. Then, we conducted an analysis of rare variants only (i.e., excluding the low-frequency V103I and I251L variants, which had the largest weight in the main analysis) to assess whether V103I and I251L were driving the association. Then, we conducted a multivariable analysis in which both *in vitro* β-arrestin recruitment and *in vitro* cAMP signaling were included in the model as possible predictors, to assess whether *in vitro* β-arrestin recruitment was an independent predictor from cAMP signaling. Then, we conducted an analysis including all 61 nonsynonymous genetic variants in *MC4R* found in European ancestry participants of UK Biobank, to assess whether the association was influenced by the focus of our main analysis on missense variants directly expressed *in vitro*. For this analysis, the level of β-arrestin recruitment of nonsense/frameshift variants of *MC4R* was assumed to be 1% of wild-type. Finally, to assess whether genotyping cluster-plot quality was influencing the association, we conducted analyses after (a) exclusion of ultra-rare genetic variants (variant allele frequency < 0.001%); (b) exclusion of variants from point (a) plus any variant with low overall cluster-plot quality score; (c) exclusion of variants from points (a-b) plus any variant with intermediate cluster-plot quality score; (d) exclusion of variants from points (a-c) plus any variant where the combined cluster-plot quality score was below 4 (ie. the maximum possible score).

External validation: We attempted to validate genetic associations using available external datasets. For 6 overlapping nonsynonymous variants in *MC4R*, we meta-analyzed our BMI-association results with those from up to 550,000 participants in the GIANT consortium (Locke et al., 2015, Turcot et al., 2018). We also used publically-accessible data from exome sequencing of 9121 type 2 diabetes cases and 9335 controls from the T2D Knowledge portal (URL: http://www.type2diabetesgenetics.org/ accessed 15th February 2019) to test the association between LoF variants in *MC4R* and type 2 diabetes.

##### Additional genetic association analyses for the V103I *MC4R* variant

The association of the V103I *MC4R* gain-of-function variant with risk of type 2 diabetes and coronary artery disease was estimated in meta-analyses of large-scale genetic association studies. The type 2 diabetes association meta-analysis included 68,906 cases and 551,079 controls from the DIAGRAM (Morris et al., 2012), EPIC-InterAct (InterAct Consortium et al., 2011) and UK Biobank (Sudlow et al., 2015) studies. The coronary artery disease association meta-analysis included 85,697 cases and 550,908 controls from the CARDIoGRAMplusC4D (Nikpay et al., 2015) and UK Biobank (Sudlow et al., 2015) studies. Association estimates from each study were combined fixed-effect inverse-variance weighted meta-analysis, as done previously with similar data sources (Lotta et al., 2018).

We also conducted a phenome-wide analysis of the association of the V103I *MC4R* variant with 353 clinical diagnoses using electronic health records from European ancestry participants of UK Biobank. Diagnoses were defined on the basis of the first three digits of ICD-10 entry codes (for instance, “F20” for a diagnosis of schizophrenia). For a given diagnosis, we considered individuals as cases if the code corresponding to the diagnosis was entered as primary or secondary diagnosis in any of their hospital admission records or as a primary or secondary cause of death in their death certificate. Individuals without the code served as controls. To minimize the burden of multiple testing and reduce the risk of false positives, we (a) considered only diagnoses with a total number of cases greater than 500, and (b) used a Bonferroni corrected p < 0.00014 (corresponding to 0.05/353) as statistical significance threshold.

#### Studies in Cellular Models

##### Functional characterization of human variants

*Cloning and site-directed mutagenesis.* MC4R cDNA constructs containing an N-terminal FLAG tag in pCDNA3.1(+) vector (Invitrogen) were used throughout the study. Site-directed mutagenesis was performed using QuickChange II XL kit (Agilent Technologies, 200516) according to the manufacturer’s protocols. All constructs were verified with Sanger sequencing. In order to characterize the functional consequences of MC4R mutants we performed assays in transiently transfected HEK293 (ATCC) and suspension HeLa cells, kindly provided by Dr Kevin Moreau (University of Cambridge).

##### Time-resolved cAMP measurement assay

Measurement of ligand-induced cAMP generation in HEK293 cells transiently expressing WT/mutant MC4R was performed using the GloSensor cAMP biosensor (Promega) according to manufacturer’s protocols. Briefly, 40,000 cells were seeded in white 96-well poly-D-lysine-coated plates. After 24 hours, cells were then transfected with both 100 ng/well of pGloSensor-20F cAMP plasmid (Promega, E1171) and 30 ng/well of plasmid encoding either WT/mutant MC4R. The day after transfection, cell media were replaced by 90 μL of fresh full DMEM with 2% v/v GloSensor cAMP Reagent (Promega, E1290) and incubated for 120 min at 37°C. Firefly luciferase activity was measured at 37°C and 5% CO_2_ using Spark 10M microplate reader (Tecan). After initial measurement of the baseline signal for 10 min (1 min intervals), cells were stimulated with 10 μL of 10x stock solution of the MC4R agonist NDP-αMSH (final concentration 1 μM) and real-time chemiluminescent signal was quantified for 45 min (30 s intervals). In each experiment, a negative control using mock transfected cells (empty pcDNA3.1(+) plasmid) and a positive control where cells were stimulated with 10 μM forskolin were assayed. The area under the curve (AUC) for cAMP production was calculated for each MC4R mutant using the baseline signal and the total peak of each curve. For data normalization, the AUC from mock transfected cells was set as 0% and the AUC from WT MC4R was set as 100%. Results represent 4-12 independent experiments. For V103I MC4R, dose-response curves using NDP-αMSH, αMSH and βMSH were plotted from total peak area under the curve values calculated for each agonist concentration, ranging from 10^−11^ to 10^−6^ M. Then, sigmoidal dose-response curves with variable slope (three-parameter logistic regression) were plotted. Normalized data were merged and presented as sum curves. Results are from 4 independent experiments.

##### Time-resolved β-arrestin recruitment assay

Coupling between MC4R and β-arrestin 2 was monitored using a NanoBiT protein: protein interaction assay (Promega, M2014). WT/mutant MC4Rs were cloned into pBiT1.1-C [TK/LgBiT] vector and β-arrestin 2 into pBiT2.1-N [TK/SmBiT] vector. Assays were performed in HEK293 cells seeded in poly-D-lysine-coated, white 96-well plates (40,000 cells/well) transiently transfected with 50 ng/well of each of the two constructs as specified previously. For the negative control, SmBiT-β-arrestin 2 construct was substituted with NanoBiT negative control vector (HaloTag-SmBiT). Positive control consisted of SmBiT-PRKACA and LgBiT-PRKAR2A vectors. Following transfection, cells were maintained overnight in cell culture medium as specified previously. The next day, 30 min prior to the assay, culture medium was substituted for 100 μl/well serum-free Opti-MEM I medium (GIBCO, 31985). Nano-luciferase activity was measured at 37°C and 5% CO_2_ using Spark 10M microplate reader (Tecan). After initial measurement of the background signal, 25 μl/well Nano-Glo® Live Cell Assay System (Promega, N2013) was added and cells were equilibrated while basal luciferase activity was measured for 10 min (1 min intervals). Subsequently, cells were stimulated with 10 μL of 13.5x stock solution of the MC4R agonist NDP-αMSH (final concentration 1 μM), and chemiluminescent signal was quantified for 45 min (30 s intervals). The area under the curve (AUC) was calculated for each MC4R mutant using the average value for the negative control as the baseline and the total peak of each curve. For data normalization, the AUC from the negative control was set as 0% and the AUC from WT MC4R was set as 100%. Results are from 4-11 independent experiments. For V103I MC4R, dose-response curves using NDP-αMSH, αMSH and βMSH were plotted from total peak area under the curve values calculated for each agonist concentration, ranging from 10^−11^ to 10^−6^ M. Then, sigmoidal dose-response curves with variable slope (three-parameter logistic regression) were plotted. Normalized data were merged and presented as sum curves. Results are from 3-5 independent experiments.

##### Western-blotting

For these experiments, 1.5 x10^5^ HEK293 cells were seeded in a poly-D-lysine coated 24-well plate and transfected the next day, when cells had reached 80% confluency, with 250 ng of MC4R constructs. Cells were serum starved overnight and stimulated for indicated periods of time with either NDP-αMSH (1 μM), αMSH (1 μM) or βMSH (1 μM). Additional stimulation with recombinant human epidermal growth factor (EGF, 10 ng/mL, Invitrogen, PHG0311, 5 min) was also performed as a positive control. Then, cells were washed once with PBS and lysed in radio-immunoprecipitation assay buffer (RIPA) (Sigma, R0278) supplemented with protease and phosphatase inhibitors (Roche cOmplete, Mini Protease Inhibitor Cocktail, 11836153001; Roche PhosSTOP, PHOSS-RO). After being harvested from the wells and centrifuged at 14,000 rpm for 15 min, the samples were prepared for electrophoresis (resuspended in 1x Bolt LDS sample buffer (Thermo, B0007) and 1x Bolt reducing agent (Thermo, B0009) and heated for 10 min at 85°C). Equal amounts of protein were used and protein electrophoresis was performed using Bolt 4%–12% Bis-Tris Plus gels (Thermo, NW04125BOX) and transfered onto nitrocellulose membrane using an iBLOT (Thermo, IB301001). After blocking with 5% bovine serum albumin (BSA) solution in Tris-buffered saline (TBS) supplemented with 0.1% Tween 20 (TBS-T) for 1 hour at room temperature, membranes were probed overnight at 4^ο^C using a Rabbit anti-p44/42 mitogen-activated protein kinase (MAPK) (extracellular signal–regulated kinases, Erk1/2) (137F5) at 1:1000 dilution (Cell Signaling Technology, 4695), a Rabbit anti-Phospho-p44/42 MAPK (Erk1/2) (Thr202/Tyr204) at 1:1000 dilution (Cell Signaling Technology, 9101) or a Rabbit-Anti-Vinculin [EPR8185] at 1:5000 dilution (Abcam, ab129002), all prepared in the blocking buffer. Cells were washed three times with TBS-T for 10 min at room temperature with gentle shaking. They were then incubated with secondary antibody, Goat anti-rabbit IgG-HRP (Dako, P0448) diluted 1:2500 in 2% BSA in TBS-T for 1 hour at room temperature. Bands were developed using enhanced chemiluminescence (ECL) substrate (Promega, W1015) and images were captured with an ImageQuant LAS 4000 (GE Healthcare). The band intensity of western blots was quantified using FIJI (Schindelin et al., 2012). For data normalization, unstimulated WT MC4R readouts were set as baseline (0%), and maximum WT MC4R ERK1/2 phosphorylation upon agonist stimulation was set as 100%. ERK1/2 phosphorylation data was plotted as pERK1/2/ tERK1/2. Results are from three to eight independent experiments.

##### ELISA for cell surface expression

Relative cell surface expression of MC4R mutants was assessed in transiently transfected HEK293 cells. 40,000 cells/well in a clear 96-well plates coated with poly-D-lysine and transfected the following day (30 ng cDNA/well). 24 hours after transfection, cells were fixed with 3.7% paraformaldehyde (15 min) at room temperature (RT) and washed three times with phosphate-buffered saline (PBS). Subsequently, non-specific binding sites were blocked with 3% non-fat dry milk in 50 mM Tris-PBS pH 7.4 (blocking buffer) for 1 hour at RT. Next, cells were incubated with a mouse monoclonal anti-FLAG (M2) antibody (Sigma-Aldrich, F1804) (dilution 1:1000 in blocking buffer) overnight at 4°C followed by triple washing with PBS and incubation with polyclonal goat anti-mouse immunoglobulins conjugated with horseradish peroxidase (HRP) (Dako, P0447) (1:1250 in 1.5% non-fat dry milk in 50 mM Tris-PBS pH 7.4) for 2 hours at RT. Finally, plates were washed three times with PBS and the high performance chromogenic substrate 3,3′,5,5′-tetramethylbenzidine (TMB CORE+, Bio-Rad Laboratories, BUF062) was used to detect HRP activity. The reaction was terminated with 0.2 M H_2_SO_4_. Color reaction product was transferred to another 96-well plate prior to measurement of absorbance at 450 nm using Infinite M1000 PRO microplate reader (Tecan). Six technical replicates were performed for each mutant in a given assay. Data were normalized to mean absorbance for pcDNA3.1(+) mock-transfected cells. Results are from four independent experiments. Statistical significance of differences between WT and mutant were estimated by unpaired t test.

##### Confocal Microscopy

150,000 HEK293 cells were seeded onto glass coverslips in 12-well plates and transfected with 250 ng of FLAG-tagged MC4R constructs. 24 h after transfection, cells were serum starved for 2 h and then stimulated for 15 min with NDP-αMSH (1 μM) at 37°C. Cells were then fixed with 4% formaldehyde in PBS for 10 min at room temperature and washed three times for 5 min with phosphate-buffered saline (PBS). After permeabilization with 0.1% Triton X-100 in PBS for 5 min, cells were incubated with phycoerythrin (PE)-conjugated anti-DYKDDDDK tag antibody, clone L5 (Biolegend, 637310) diluted 1:100 in blocking buffer. Slides were imaged using a Leica SP8 confocal microscope and images processed using FIJI. Results are from three independent experiments.

##### Fluorescence-activated cell sorting (FACS)

80,000 suspension HeLa cells were seeded in 6-well CytoOne plates (USA Scientific, CC7672-7506) and transfected with 250 ng of FLAG-tagged MC4R constructs. 24 hours after transfection, cells were serum starved for 2 hours and then stimulated for 15 min with NDP-αMSH (1 μM) at 37°C. Cells were washed once with serum-free medium, high glucose DMEM, no phenol red (DMEM, GIBCO, 31053), followed by fixation with 4% formaldehyde in PBS for 10 min at room temperature. After washing three times with PBS, cells were incubated at 4°C for 30 min with PE-conjugated anti-DYKDDDDK tag antibody, clone L5 (Biolegend, 637310) diluted 1:100 in serum-free medium. After an extra wash step, cells were analyzed on FACS Accuri C6 (BD Biosciences). Flow cytometry data analysis and mean fluorescence intensity (MFI) values were calculated by FlowJo analysis software (Tree Star) on live-gated cells (minimum of 20,000 cells). Percent internalization was calculated based on MFI values as follows: % internalization = (1 – MFI NDP-αMSH stimulated/MFI unstimulated) x 100. Results are from four independent experiment.

### Quantification and Statistical Analysis

Results were analyzed using GraphPad Prism 7 (Graph Pad Software), STATA v14.2 (StataCorp, College Station, Texas 77845 USA), R v3.2.2 (The R Foundation for Statistical Computing). For cAMP, β-arrestin and ERK1/2 assays, the difference between WT and mutant MC4Rs was tested using an unpaired single-sample t test assigning a value of 100% for WT. The signaling bias for GoF variants of *MC4R* was estimated by calculating the ratio of geometric means for E_max_ β-arrestin to E_max_ cAMP and its 95% confidence interval using unpaired two-sample t test, with a null hypothesis of no bias (i.e., ratio = 1). Studies in cellular models are from at least 3 independent experiments. All p values reported in this manuscript are from 2-sided statistical tests. A p < 0.05 was considered statistically significant. In figures, statistical significance is represented as ^∗^p < 0.05, ^∗∗^p < 0.01 and ^∗∗∗^p < 0.001.

## Supplemental Information

Table S1. Characteristics of European-Ancestry Participants of UK Biobank and of the Nonsynonymous Genetic Variants in *MC4R* Included in This Study, Related to Figure 1

Table S2. Functional Characterization of *MC4R* Variants Identified in UK Biobank, Related to Figure 1

Table S3. Association of Gain-of-Function and Loss-of-Function Variants in *MC4R* with Blood Pressure and Resting Heart Rate, Related to Figure 1

Table S4. Associations with Body Mass Index in Sensitivity and Validation Analyses, Related to Figure 1

Table S5. Meta-regression Analyses Investigating whether *In Vitro* cAMP Production or β-Arrestin Recruitment Explain Variance in the Associations of *MC4R* Variants with BMI, Related to Figure 2

Table S6. Associations of cAMP-Biased Gain-of-Function Variants in *MC4R* with Continuous Traits and Disease Outcomes, Related to Figure 3

Table S7. Association of the Gain-of-Function Variant V103I *MC4R* with Continuous Traits and Disease Outcomes Related to Figures 3 and 4

Table S8. Functional Characterization of V103I MC4R, Related to Figure 4
