# Supplementary material for: Human Gain-of-Function MC4R Variants Show Signaling Bias and Protect against Obesity
Source: Cell. 2019 Apr 18;177(3):597–607.e9. doi: 10.1016/j.cell.2019.03.044 (PMC6476272; doi:10.1016/j.cell.2019.03.044)
Supplement: Table S1. Characteristics of European-Ancestry Participants of UK Biobank and of the Nonsynonymous Genetic Variants in MC4R Included in This Study, Related to Figure 1 [file mmc1.pdf]

Cell, Volume *177*

Supplemental Information

Human Gain-of-Function *MC4R* Variants

Show Signaling Bias and Protect against Obesity

Luca A. Lotta, Jacek Mokrosiński, Edson Mendes de Oliveira, Chen Li, Stephen J. Sharp, Jian'an Luan, Bas Brouwers, Vikram Ayinampudi, Nicholas Bowker, Nicola Kerrison, Vasileios Kaimakis, Diana Hoult, Isobel D. Stewart, Eleanor Wheeler, Felix R. Day, John R.B. Perry, Claudia Langenberg, Nicholas J. Wareham, and I. Sadaf Farooqi

Table S1. Characteristics of European ancestry participants of UK Biobank and the nonsynonymous genetic variants in *MC4R* included in this study. Related to Figure 1.

Study	UK Biobank
Country	United Kingdom
Genotyping chip	Affymetrix UK BILEVE and UK Biobank Axiom arrays
Imputation panels	Haplotype Reference Consortium r1.1, 1000 Genomes phase 3 and UK10K
Participants, n	452,300
Female sex, n (%)	245,335 (54)
Age, mean (SD) in years	57 (8)
Current smokers at baseline, n (%)	47,036 (10)
BMI, mean (SD) in kg/m^2	27.4 (4.8)
Systolic blood pressure, mean (SD) in mmHg	138 (19)
Diastolic blood pressure, mean (SD) in mmHg	82 (10)
Resting heart rate, mean (SD) in bpm	70 (12)

Genomic coordinates, chromosome and position	Reference allele	Variant allele	Protein change	Genotyped or imputed (imputation quality info score)	Variant allele frequency
Chr18:58039563	C	T	R7H	Genotyped	0.006%
Chr18:58039551	G	C	T11S	Genotyped	0.003%
Chr18:58039535	C	T	W16X	Genotyped	0.0004%
Chr18:58039494	G	A	S30F	Genotyped	0.02%
Chr18:58039483	C	CT	G34RfsX4	Genotyped	0.004%
Chr18:58039478	G	T	Y35X[a]	Genotyped	0.007%
Chr18:58039456	G	A	Q43X	Genotyped	0.001%
Chr18:58039435	C	T	V50M	Genotyped	0.0006%
Chr18:58039419	C	T	G55D	Genotyped	0.002%
Chr18:58039356	T	C	H76R	Genotyped	0.006%
Chr18:58039350	G	A	P78L	Genotyped	0.0003%
Chr18:58039346	C	G	M79I	Genotyped	0.002%
Chr18:58039343	G	T	Y80X	Genotyped	0.002%
Chr18:58039315	C	T	D90N	Genotyped	0.001%
Chr18:58039304	CACCAGCATATCAGC	-	A89_V93del	Genotyped	0.0004%
Chr18:58039302	C	T	S94N	Genotyped	0.001%
Chr18:58039300	C	T	V95I	Genotyped	0.009%
Chr18:58039281	G	T	T101N	Genotyped	0.001%
Chr18:58039276	C	T	V103I	Genotyped	2.0%
Chr18:58039266	A	G	L106P	Genotyped	0.0002%
Chr18:58039248	G	A	T112M	Imputed (0.82)	0.07%
Chr18:58039236	CT	-	S116FfsX6	Genotyped	0.0007%
Chr18:58039177	A	G	S136P	Genotyped	0.001%
Chr18:58039134	G	A	T150I	Genotyped	0.003%
Chr18:58039133	AGTAA	-	F149YfsX9	Genotyped	0.002%
Chr18:58039122	G	T	A154D	Genotyped	0.002%
Chr18:58039098	G	A	T162I	Genotyped	0.002%
Chr18:58039090	G	A	R165W	Genotyped	0.004%
Chr18:58039089	C	T	R165Q	Genotyped	0.004%
Chr18:58039087	C	T	V166I	Genotyped	0.003%
Chr18:58039060	C	T	A175T	Genotyped	0.002%
Chr18:58038983	CAT	-	M200del	Genotyped	0.002%
Chr18:58038980	G	T	F201L	Genotyped	0.001%
Chr18:58038977	G	T	F202L	Genotyped	0.002%
Chr18:58038951	A	G	L211P	Genotyped	0.0002%
Chr18:58038927	G	A	A219V	Genotyped	0.0002%
Chr18:58038892	C	T	G231S	Genotyped	0.001%
Chr18:58038891	C	A	G231V	Genotyped	0.004%
Chr18:58038877	G	A	R236C	Genotyped	0.01%
Chr18:58038870	C	T	G238D	Genotyped	0.002%
Chr18:58038852	G	T	A244E	Genotyped	0.006%
Chr18:58038832	T	G	I251L	Imputed (0.90)	1.3%
Chr18:58038829	C	T	G252S	Genotyped	0.02%
Chr18:58038826	C	T	V253I	Genotyped	0.01%
Chr18:58038801	A	G	F261S	Genotyped	0.001%

Table S1. Continued.

Genomic coordinates, chromosome and position	Reference allele	Variant allele	Protein change	Genotyped or imputed (imputation quality info score)	Variant allele frequency
Chr18:58038777	A	T	I269N	Genotyped	0.004%
Chr18:58038771	C	A	C271F	Genotyped	0.004%
Chr18:58038760	G	A	P275S	Genotyped	0.0003%
Chr18:58038746	G	GCA	F280AfsX12	Genotyped	0.006%
Chr18:58038722	A	T	Y287X	Genotyped	0.001%
Chr18:58038718	T	A	I289L	Genotyped	0.002%
Chr18:58038681	A	G	I301T	Genotyped	0.0004%
Chr18:58038678	T	A	Y302F	Genotyped	0.004%
Chr18:58038676	C	T	A303T	Genotyped	0.0008%
Chr18:58038673	G	A	L304F	Genotyped	0.0001%
Chr18:58038664	G	A	Q307X	Genotyped	0.002%
Chr18:58038654	C	T	R310K	Genotyped	0.0008%
Chr18:58038636	A	C	I316S	Genotyped	0.002%
Chr18:58038634	T	C	I317V	Genotyped	0.004%
Chr18:58038610	G	A	L325F	Genotyped	0.002%
Chr18:58038588	T	C	Y332C	Genotyped	0.002%

Upper part; n, number of participants; BMI, body mass index; SD, standard deviation; mmHg, millimetres of mercury (mmHg); bpm, beats per minute. Lower part; Chromosome and coordinate positions are according to human genome reference sequence hg19/build37. Protein changes are on the basis of transcript NCBI messenger RNA Reference Sequence NM_005912. The imputation info score is a measure of the quality of imputation, where a value of 1 indicates perfect imputation or direct genotyping. Variant allele frequencies are from European ancestry participants of UK Biobank included in this study. [a] Y35X occurs as a haplotype with D37V.
