# Supplementary material for: Human Gain-of-Function MC4R Variants Show Signaling Bias and Protect against Obesity
Source: Cell. 2019 Apr 18;177(3):597–607.e9. doi: 10.1016/j.cell.2019.03.044 (PMC6476272; doi:10.1016/j.cell.2019.03.044)
Supplement: Table S2. Functional Characterization of MC4R Variants Identified in UK Biobank, Related to Figure 1 [file mmc2.pdf]

Table S2. Functional characterization of *MC4R* variants identified in UK Biobank. Related to Figure 1.

MC4R variant	Cell surface expression					cAMP production					β-arrestin 2 recruitment				
	Mean	Lower 95% CI of mean	Upper 95% CI of mean	n	*P* value	Mean	Lower 95% CI of mean	Upper 95% CI of mean	n	*P* value	Mean	Lower 95% CI of mean	Upper 95% CI of mean	n	*P* value
WT	100	n/a	n/a	4	n/a	100	n/a	n/a	12	n/a	100	n/a	n/a	15	n/a
R7H	128.6	107.3	149.8	4	0.08	116.2	97.40	135.1	7	0.08	54.07	43.08	65.06	5	<0.001
T11S	111.1	97.44	124.7	4	0.02	139.9	119.7	160.1	7	0.003	82.86	55.20	110.5	4	0.14
S30F	106.7	75.17	138.1	4	0.55	108.7	91.72	125.8	4	0.20	108.5	94.07	122.9	4	0.16
V50M	124.4	71.87	176.9	4	0.24	118.9	95.75	142.0	5	0.09	26.99	18.18	35.80	4	<0.001
G55D	66.20	33.18	99.22	4	0.05	46.86	27.49	66.23	12	<0.001	0.9601	<0.1	1.846	4	<0.001
H76R	66.29	39.45	93.12	4	0.03	88.42	68.05	108.8	4	0.17	10.92	6.235	15.60	4	<0.001
P78L	52.67	19.92	85.42	4	0.02	0.3821	<0.1	1.039	5	<0.001	<0.1	<0.1	<0.1	4	<0.001
M79I	61.47	37.30	85.63	4	0.01	51.55	35.11	67.98	4	0.003	39.90	27.23	52.57	6	<0.001
D90N	77.06	46.99	107.1	4	0.09	1.794	1.167	2.420	9	<0.001	7.963	3.714	12.21	4	<0.001
S94N	87.05	37.84	136.3	4	0.46	62.12	36.75	87.49	12	0.007	0.6295	0.1022	1.157	4	<0.001
V95I	52.15	23.74	80.56	4	0.01	82.60	55.41	109.8	5	0.15	14.10	5.547	22.65	4	<0.001
T101N	129.5	81.34	177.7	4	0.15	153.4	121.2	185.5	4	0.01	65.24	56.64	73.84	6	<0.001
V103I	113.9	66.46	161.3	4	0.42	168.1	137.1	199.0	4	0.006	278.3	210.9	345.7	7	0.001
L106P	52.21	26.32	78.09	4	0.01	76.19	57.35	95.02	8	0.02	11.88	8.188	15.57	4	<0.001
T112M	114.1	105.3	122.8	4	0.01	125.5	98.21	152.8	7	0.06	66.13	45.87	86.39	4	0.01
S136P	74.60	63.67	85.54	4	0.005	0.4757	<0.1	1.283	5	<0.001	<0.1	<0.1	<0.1	4	<0.001
T150I	71.47	69.23	73.71	4	<0.001	36.08	24.11	48.06	5	<0.001	53.09	42.94	63.24	4	0.001
A154D	81.23	65.34	97.12	4	0.03	109.1	83.38	134.9	12	0.45	38.80	24.91	52.69	5	<0.001
T162I	62.08	56.75	67.40	4	<0.001	6.695	1.331	12.06	5	<0.001	<0.1	<0.1	<0.1	4	<0.001
R165Q	55.69	37.47	73.91	4	0.005	62.82	48.89	76.76	7	0.001	<0.1	<0.1	<0.1	4	<0.001
R165W	60.32	28.60	92.04	4	0.03	83.10	58.11	108.1	7	0.15	0.1705	<0.1	0.2862	4	<0.001
V166I	105.0	80.53	129.4	4	0.56	118.5	88.13	148.9	7	0.19	11.61	8.076	15.14	4	<0.001
A175T	51.78	24.65	78.92	4	0.01	55.56	45.37	65.75	4	0.001	34.73	26.56	42.90	4	<0.001
F201L	102.1	38.35	165.9	4	0.92	158.5	133.4	183.6	4	0.005	95.83	66.54	125.1	7	0.74
F202L	99.54	74.73	124.3	4	0.96	102.8	81.55	124.0	4	0.70	53.34	36.42	70.26	8	<0.001
L211P	77.43	22.11	132.7	4	0.28	17.00	8.475	25.52	9	<0.001	0.1037	<0.1	0.2229	4	<0.001
A219V	63.32	26.92	99.71	4	0.05	28.99	10.28	47.71	5	<0.001	100.5	78.41	122.6	6	0.96
G231S	85.24	52.20	118.3	4	0.25	168.7	150.8	186.5	4	0.001	84.17	60.53	107.8	6	0.15
G231V	111.4	72.78	150.1	4	0.42	102.4	73.48	131.4	7	0.85	118.6	90.65	146.5	7	0.15
R236C	120.7	75.42	165.9	4	0.24	139.3	103.4	175.3	7	0.04	34.06	19.11	49.01	7	<0.001
G238D	108.6	85.40	131.7	4	0.32	92.07	56.56	127.6	7	0.60	104.4	70.14	138.7	10	0.78
A244E	98.31	78.83	117.8	4	0.80	69.38	43.54	95.23	8	0.03	11.06	1.722	20.40	6	<0.001
I251L	111.1	87.43	134.9	4	0.23	124.0	96.68	151.3	4	0.07	209.7	163.4	256.0	4	0.005
G252S	55.23	29.22	81.23	4	0.01	49.58	34.99	64.17	4	0.002	40.54	31.07	50.01	6	<0.001
V253I	78.52	39.11	117.9	4	0.18	107.6	96.44	118.8	4	0.12	21.68	5.435	37.93	6	<0.001
F261S	89.53	84.12	94.95	4	0.009	85.57	47.52	123.6	8	0.40	0.6371	0.2668	1.007	4	<0.001
I269N	<0.1	<0.1	1.041	4	<0.001	0.3243	<0.1	0.8696	5	<0.001	2.326	0.3147	4.337	4	<0.001
C271F	99.87	82.65	117.1	4	0.98	24.77	11.13	38.41	5	<0.001	<0.1	<0.1	<0.1	4	<0.001
P275S	102.7	85.23	120.1	4	0.66	55.12	43.06	67.18	4	0.001	66.37	52.79	79.95	6	0.001
I289L	99.01	90.07	107.9	4	0.75	92.96	79.80	106.1	4	0.19	233.7	190.7	276.7	4	0.002
I301T	78.21	67.33	89.08	4	0.008	30.23	11.55	48.90	5	<0.001	0.1668	<0.1	0.2524	4	<0.001
Y302F	63.15	43.14	83.16	4	0.01	90.08	68.64	111.5	4	0.24	50.67	35.78	65.56	10	<0.001
A303T	62.67	55.52	69.82	4	<0.001	12.51	8.849	16.16	5	<0.001	26.95	13.77	40.13	6	<0.001
L304F	109.3	79.93	138.7	4	0.39	121.7	109.6	133.9	4	0.01	144.3	112.1	176.5	11	0.01
R310K	93.34	16.88	169.8	4	0.80	87.60	74.30	100.9	4	0.06	14.15	5.034	23.27	4	<0.001
I316S	94.06	74.98	113.1	4	0.39	64.63	43.79	85.47	8	0.005	1.047	0.3879	1.706	4	<0.001
I317V	103.8	87.85	119.8	4	0.50	125.5	100.0	151.0	6	0.05	196.4	135.1	257.7	7	0.008
L325F	109.5	72.40	146.5	4	0.48	105.9	89.06	122.8	4	0.35	41.40	36.22	46.58	5	<0.001
Y332C	91.41	79.18	103.6	4	0.11	137.5	114.8	160.3	6	0.008	147.0	98.27	195.7	11	0.06

Characterization of 49 MC4R variants: cell surface expression measured by ELISA and maximal effect of NDP-αMSH-induced cAMP production and β-arrestin recruitment measured using time-resolved assays. CI, confidence interval; n, number of independent experiments; n/a, non-applicable.
