# Supplementary material for: Human Gain-of-Function MC4R Variants Show Signaling Bias and Protect against Obesity
Source: Cell. 2019 Apr 18;177(3):597–607.e9. doi: 10.1016/j.cell.2019.03.044 (PMC6476272; doi:10.1016/j.cell.2019.03.044)
Supplement: Table S3. Association of Gain-of-Function and Loss-of-Function Variants in MC4R with Blood Pressure and Resting Heart Rate, Related to Figure 1 [file mmc3.pdf]

Table S3. Association of gain-of-function and loss-of-function variants in *MC4R* with blood pressure and resting heart rate. Related to Figure 1.

Outcome	n	Exposure	Number of variants	Outcome unit	Beta (95% CI)	*P* value
Systolic blood pressure	451,439	Gain-of-Function variants	9	mmHg	0.03 (-0.17, 0.23)	0.78
		Loss-of-Function variants	47		-0.24 (-1.05, 0.58)	0.57
Diastolic blood pressure	451,452	Gain-of-Function variants	9	mmHg	-0.13 (-0.25, -0.01)	0.03
		Loss-of-Function variants	47		0.23 (-0.24, 0.70)	0.34
Resting heart rate	422,425	Gain-of-Function variants	9	bpm	-0.20 (-0.34, -0.07)	0.003
		Loss-of-Function variants	47		-0.38 (-0.93, 0.17)	0.17

Association analyses were conducted in European ancestry participants of UK Biobank and individual variant estimates were pooled using inverse-variance weighted meta-analysis. Beta coefficients and their 95% confidence intervals are in units of outcome per allele. n, number of participants; CI, confidence interval; mmHg, millimetres of mercury; bpm, beats per minute.
