# Supplementary material for: Human Gain-of-Function MC4R Variants Show Signaling Bias and Protect against Obesity
Source: Cell. 2019 Apr 18;177(3):597–607.e9. doi: 10.1016/j.cell.2019.03.044 (PMC6476272; doi:10.1016/j.cell.2019.03.044)
Supplement: Table S4. Associations with Body Mass Index in Sensitivity and Validation Analyses, Related to Figure 1 [file mmc4.pdf]

Table S4. Associations with Body Mass Index in sensitivity and validation analyses. Related to Figure 1.

Exposure	Exclusion criteria	Variants, n	Beta (SE)	*P* value
GoF variants	MAF < 0.001%[a]	7	-0.378 (0.026)	2×10^{-47}
LoF variants		33	0.260 (0.108)	0.02
GoF variants	MAF < 0.001%[a] or low-cluster-plot-quality score[b]	7	-0.378 (0.026)	2×10^{-47}
LoF variants		32	0.273 (0.109)	0.01
GoF variants	MAF < 0.001%[a] or low or intermediate-cluster-plot-quality score[c]	7	-0.378 (0.026)	2×10^{-47}
LoF variants		30	0.294 (0.110)	0.008
GoF variants	MAF < 0.001% [a] or cluster-plot-quality score below 4 (i.e. highest possible score)[d]	7	-0.378 (0.026)	2×10^{-47}
LoF variants		23	0.284 (0.123)	0.02
Complete LoF variants of either pathway [e]	Partial LoF variants	26	0.722 (0.196)	0.0002
Complete LoF variants of either pathway [e, f]	Partial LoF variants	26	1.090 (0.180)	1×10^{-9}

Study	Participants, n	Variant	Variant allele frequency[g]	Beta (95% CI)[h]	*P* value	*P* –heterogeneity[i]
UK Biobank	450,708	V103I	2.0%	-0.52 (-0.58, -0.45)	1×10^{-54}	0.14
GIANT	287,276			-0.42 (-0.53, -0.31)	1×10^{-13}	
Meta-analysis	737,984			-0.49 (-0.55, -0.44)	3×10^{-66}	
UK Biobank	450,708	I251L	1.3%	-0.15 (-0.24, -0.07)	4×10^{-4}	0.94
GIANT	526,508			-0.16 (-0.25, -0.07)	7×10^{-4}	
Meta-analysis	977,216			-0.15 (-0.22, -0.09)	9×10^{-7}	
UK Biobank	450,708	F202L	0.002%	-0.91 (-3.15, 1.32)	0.42	0.89
GIANT	526,508			-0.62 (-4.17, 2.93)	0.73	
Meta-analysis	977,216			-0.83 (-2.72, 1.06)	0.39	
UK Biobank	450,708	I269N	0.004%	0.71 (-0.69, 2.11)	0.32	0.78
GIANT	526,508			1.34 (-2.87, 5.54)	0.53	
Meta-analysis	977,216			0.77 (-0.56, 2.10)	0.26	
UK Biobank	450,708	A175T	0.002%	-0.69 (-2.54, 1.15)	0.46	0.47
GIANT	526,508			0.02 (-0.65, 0.70)	0.95	
Meta-analysis	977,216			-0.06 (-0.69, 0.57)	0.85	
UK Biobank	450,708	Y35X	0.007%	1.85 (0.78, 2.92)	0.0007	0.09
GIANT	526,508			3.05 (2.16, 3.95)	2×10^{-11}	
Meta-analysis	977,216			2.56 (1.87, 3.24)	3×10^{-13}	

Abbreviations: BMI, body mass index; Beta in kg/m^2 per allele; GoF, gain-of-function; LoF, loss-of-function; MAF, minor allele frequency; UK, United Kingdom; GIANT, Genetic investigation of anthropometric traits consortium; n, number of participants; SE, standard error; CI, Confidence interval.

[a] Excluded variants: GoF, L304F, G231S; LoF, R310K, A303T, I301T, P275S, A219V, L211P, L106P, S94N, D90N, P78L, V50M, A89_V93del, W16X, S116FfsX6.
[b] Excluded variant: LoF, V166I.
[c] Excluded variants: LoF, V166I, F202L, G55D.
[d] Excluded variants: LoF, V166I, F202L, G55D, Y302F, I269N, F261S, V253I, T162I, T150I, F280AfsX12.
[e] These analyses included only nonsense or frameshift variants or variants where cAMP production or beta-arrestin recruitment were <5% of wild-type *in vitro*.
[f] Based on a meta-analysis of UK Biobank and GIANT.
[g] In UK Biobank.
[h] In kg/m^2 of BMI per copy of the variant allele.
[i] *P* value for heterogeneity in association estimates between UK Biobank and GIANT.
