# Supplementary material for: Human Gain-of-Function MC4R Variants Show Signaling Bias and Protect against Obesity
Source: Cell. 2019 Apr 18;177(3):597–607.e9. doi: 10.1016/j.cell.2019.03.044 (PMC6476272; doi:10.1016/j.cell.2019.03.044)
Supplement: Table S5. Meta-regression Analyses Investigating whether In Vitro cAMP Production or β-Arrestin Recruitment Explain Variance in the Associations of MC4R Variants with BMI, Related to Figure 2 [file mmc5.pdf]

Table S5. Meta-regression analyses investigating whether *in vitro* cAMP production or β-arrestin recruitment explain variance in associations of *MC4R* variants with BMI. Related to Figure 2.

Analysis	Excluded variant(s)	Number of variants	Slope (95% CI)	*P* value
Leave-one-out [a]	R7H	48	-0.37 (-0.53, -0.21)	3×10^{-05}
Leave-one-out	T11S	48	-0.37 (-0.53, -0.21)	2×10^{-05}
Leave-one-out	S30F	48	-0.37 (-0.53, -0.21)	4×10^{-05}
Leave-one-out	V50M	48	-0.37 (-0.53, -0.21)	3×10^{-05}
Leave-one-out	G55D	48	-0.38 (-0.54, -0.22)	2×10^{-05}
Leave-one-out	H76R	48	-0.36 (-0.52, -0.20)	4×10^{-05}
Leave-one-out	P78L	48	-0.39 (-0.55, -0.23)	9×10^{-06}
Leave-one-out	M79I	48	-0.37 (-0.53, -0.21)	3×10^{-05}
Leave-one-out	D90N	48	-0.37 (-0.53, -0.21)	2×10^{-05}
Leave-one-out	S94N	48	-0.36 (-0.52, -0.20)	4×10^{-05}
Leave-one-out	V95I	48	-0.36 (-0.52, -0.20)	4×10^{-05}
Leave-one-out	T101N	48	-0.37 (-0.53, -0.21)	2×10^{-05}
Leave-one-out	V103I	48	-0.33 (-0.47, -0.19)	2×10^{-05}
Leave-one-out	L106P	48	-0.36 (-0.52, -0.21)	3×10^{-05}
Leave-one-out	T112M	48	-0.36 (-0.52, -0.20)	5×10^{-05}
Leave-one-out	S136P	48	-0.34 (-0.51, -0.17)	0.0002
Leave-one-out	T150I	48	-0.37 (-0.53, -0.21)	3×10^{-05}
Leave-one-out	A154D	48	-0.37 (-0.53, -0.21)	3×10^{-05}
Leave-one-out	T162I	48	-0.45 (-0.61, -0.30)	3×10^{-07}
Leave-one-out	R165W	48	-0.38 (-0.55, -0.21)	3×10^{-05}
Leave-one-out	R165Q	48	-0.32 (-0.49, -0.15)	0.0003
Leave-one-out	V166I	48	-0.37 (-0.53, -0.21)	2×10^{-05}
Leave-one-out	A175T	48	-0.37 (-0.53, -0.21)	3×10^{-05}
Leave-one-out	F201L	48	-0.37 (-0.53, -0.21)	3×10^{-05}
Leave-one-out	F202L	48	-0.37 (-0.53, -0.21)	3×10^{-05}
Leave-one-out	L211P	48	-0.37 (-0.53, -0.21)	2×10^{-05}
Leave-one-out	A219V	48	-0.37 (-0.53, -0.21)	3×10^{-05}
Leave-one-out	G231S	48	-0.37 (-0.53, -0.21)	3×10^{-05}
Leave-one-out	G231V	48	-0.37 (-0.53, -0.21)	3×10^{-05}
Leave-one-out	R236C	48	-0.36 (-0.52, -0.21)	2×10^{-05}
Leave-one-out	G238D	48	-0.37 (-0.53, -0.21)	3×10^{-05}
Leave-one-out	A244E	48	-0.37 (-0.53, -0.21)	3×10^{-05}
Leave-one-out	I251L	48	-0.38 (-0.55, -0.21)	5×10^{-05}
Leave-one-out	G252S	48	-0.36 (-0.52, -0.20)	4×10^{-05}
Leave-one-out	V253I	48	-0.37 (-0.53, -0.20)	4×10^{-05}
Leave-one-out	F261S	48	-0.35 (-0.50, -0.19)	4×10^{-05}
Leave-one-out	I269N	48	-0.36 (-0.53, -0.20)	5×10^{-05}
Leave-one-out	C271F	48	-0.31 (-0.48, -0.13)	0.001
Leave-one-out	P275S	48	-0.37 (-0.53, -0.21)	3×10^{-05}
Leave-one-out	I289L	48	-0.37 (-0.53, -0.21)	2×10^{-05}
Leave-one-out	I301T	48	-0.38 (-0.54, -0.22)	1×10^{-05}
Leave-one-out	Y302F	48	-0.37 (-0.53, -0.21)	3×10^{-05}
Leave-one-out	A303T	48	-0.37 (-0.53, -0.21)	3×10^{-05}
Leave-one-out	L304F	48	-0.37 (-0.52, -0.21)	3×10^{-05}
Leave-one-out	R310K	48	-0.36 (-0.52, -0.21)	3×10^{-05}
Leave-one-out	I316S	48	-0.37 (-0.53, -0.21)	3×10^{-05}
Leave-one-out	I317V	48	-0.37 (-0.53, -0.21)	3×10^{-05}
Leave-one-out	L325F	48	-0.37 (-0.53, -0.21)	3×10^{-05}
Leave-one-out	Y332C	48	-0.36 (-0.51, -0.21)	2×10^{-05}
Rare variants only [b]	V103I and I251L	47	-0.32 (-0.51, -0.13)	0.001
Multivariable model with cAMP signaling as co-variate [c]	None	49	-0.41 (-0.61, -0.20)	0.0003
Truncating variants included [d]	None	61	-0.32 (-0.48, -0.16)	0.0002
Ultra-rare variants excluded [e]	13 variants [e]	36	-0.40 (-0.57, -0.23)	3×10^{-05}
Ultra-rare or low-cluster-plot-quality score excluded [f]	14 variants [f]	35	-0.41 (-0.58, -0.24)	2×10^{-05}
Ultra-rare or low- or intermediate-cluster-plot-quality score excluded [g]	16 variants [g]	33	-0.42 (-0.60, -0.25)	2×10^{-05}
Ultra-rare or cluster-plot-quality score below 4 excluded [h]	24 variants [h]	25	-0.52 (-0.69, -0.35)	2×10^{-06}
All missense variants (main analysis) [i]	None	49	-0.37 (-0.52, -0.21)	3×10^{-05}

Predictor (Co-variate)	Outcome	Number of variants	Slope (95% CI)	*P* value
cAMP production (None)	BMI association estimates	49 missense variants	-0.31 (-0.78, 0.16)	0.19
cAMP production (β-arrestin recruitment)		49 missense variants	0.16 (-0.34, 0.67)	0.52
β-arrestin recruitment (None)		20 missense variants which were wild-type-like for cAMP production	-0.32 (-0.59, -0.06)	0.02
β-arrestin functional category [j] (None)		49 missense variants	-0.31 (-0.59, -0.02) [j]	0.04 [j]

Table S5. Continued.

Upper part; Sensitivity meta-regression analyses investigating whether in vitro β-arrestin recruitment explains the variance in associations of MC4R variants with BMI. The potential for β-arrestin recruitment (predictor) to explain the variance (i.e. between-genetic-variants variance) in the association of MC4R variants with BMI (outcome) was investigated using random-effects meta-regression. A total of 56 sensitivity analyses are presented in the panel. [a)] 49 leave-one-out analyses where each missense variant was excluded at a given iteration. [b)] An analysis of rare variants only (i.e. excluding the low-frequency V103I and I251L variants, which had the largest weight in the main analysis). [c)] A multivariable analysis in which both β-arrestin recruitment and cAMP production were included in the model as possible predictors. [d)] An analysis including all 61 nonsynonymous variants in MC4R found in European ancestry participants of UK Biobank. For this analysis, the level of β-arrestin recruitment of nonsense/frameshift MC4R variants was assumed to be 1% of wild-type. [e)] An analysis where variants with minor allele frequency <0.001% were excluded: R310K, L304F, A303T, I301T, P275S, G231S, A219V, L211P, L106P, S94N, D90N, P78L, V50M. [f)] An analysis where variants with minor allele frequency <0.001% or variants with low cluster-plot quality score were excluded: variants listed in note [e] plus V166I. [g)] An analysis where variants with minor allele frequency <0.001% or variants with low or intermediate cluster-plot quality score were excluded: variants listed in notes [e-f] plus F202L, G55D. [h)] An analysis where variants with minor allele frequency <0.001% or variants with cluster-plot quality score below 4 were excluded: variants listed in notes [e-g] plus Y302F, I269N, F261S, V253I, G238D, G231V, T162I, T150I. [i)] The main analysis included all 49 missense variants in *MC4R* found in European ancestry participants of UK Biobank (presented in Fig. 2 and also reported at the bottom of this table to facilitate comparisons). Lower part; Meta-regression analyses investigating whether cAMP production explains the variance in associations of *MC4R* variants with BMI. The potential for cAMP (predictor) production to explain the variation (i.e. between-genetic-variants variance) in the association of the 49 missense MC4R variants with BMI (outcome) was investigated using random-effects meta-regression. Two meta-regression analyses are reported. The first included cAMP production as the only predictor. The second was a multivariable model including β-arrestin recruitment as an additional predictor (covariate). Also, a meta-regression analysis was conducted investigating whether β-arrestin recruitment explains the variance in associations with BMI of *MC4R* variants that were wild-type like for cAMP production. This analysis included 20 missense variants that were wild-type-like for cAMP production. The slope describes the linear relationship between the predictor (expressed in natural-logarithm-transformed ratio of variant allele to wild-type) and BMI associations (outcome; estimated in UK Biobank and expressed in kg/m^2 per allele) for the number of missense variants. Also, an analysis was conducted using the functional category of β-arrestin recruitment as predictor. [j)] The predictor was whether a variant was a loss-of-function, wild-type-like or gain-of-function variant for β-arrestin (coded as 0, 1, 2 respectively), the slope is in kg/m^2 per allele for a unit increase in functional category (ie. From LoF to WT-like or from WT-like to GoF). *P*-value for association of functional category was *P*=0.54 after excluding LoF variants and *P*=0.54 after excluding GoF variants in two distinct sensitivity analyses. cAMP, cyclic adenosine monophosphate; BMI, body mass index; CI, confidence interval.
