# Supplementary material for: Human Gain-of-Function MC4R Variants Show Signaling Bias and Protect against Obesity
Source: Cell. 2019 Apr 18;177(3):597–607.e9. doi: 10.1016/j.cell.2019.03.044 (PMC6476272; doi:10.1016/j.cell.2019.03.044)
Supplement: Table S6. Associations of cAMP-Biased Gain-of-Function Variants in MC4R with Continuous Traits and Disease Outcomes, Related to Figure 3 [file mmc6.pdf]

Table S6. Associations of cAMP-biased gain-of-function variants in *MC4R* with continuous traits and disease outcomes. Related to Figure 3.

Genotype	Outcome, unit	n	Median value (IQR) in outcome units	Beta in outcome units (95% CI)	*P* value
Non-carriers	BMI, kg/m^2	450,503	26.7 (24.1, 29.9)	Reference	Reference
Carriers of a cAMP biased GoF variant		166	27.1 (24.7, 31.1)	0.79 (0, 1.58)	0.05
Non-carriers	Systolic blood pressure, mmHg	451,234	138 (126, 153)	Reference	Reference
Carriers of a cAMP biased GoF variant		166	142 (130, 156)	3.1 (0.5, 5.7)	0.02
Non-carriers	Diastolic blood pressure, mmHg	451,247	83 (76, 91)	Reference	Reference
Carriers of a cAMP biased GoF variant		166	85 (77, 91)	1.4 (-0.1, 3.0)	0.07
Non-carriers	Resting heart rate, bpm	422,232	68 (62, 76)	Reference	Reference
Carriers of a cAMP biased GoF variant		154	68 (61, 76)	-0.5 (-2.2, 1.3)	0.61
**Genotype**	**Outcome**	**n**	**Percentage of people with disease in genotype category (N)**	**Odds ratio (95% CI)**	***P* value**
Non-carriers	Obesity	258,620	42% (109,076)	Reference	Reference
Carriers of a cAMP biased GoF variant		101	52% (53)	1.45 (0.95, 2.19)	0.08
Non-carriers	Severe obesity	158,035	5% (8,491)	Reference	Reference
Carriers of a cAMP biased GoF variant		52	8% (4)	1.46 (0.52, 4.06)	0.47
Non-carriers	Type 2 diabetes	449,130	6% (24,744)	Reference	Reference
Carriers of a cAMP biased GoF variant		166	7% (12)	1.23 (0.67, 2.23)	0.50
Non-carriers	Coronary artery disease	452,096	6% (24,883)	Reference	Reference
Carriers of a cAMP biased GoF variant		167	5% (9)	0.87 (0.43, 1.76)	0.71

Associations with continuous traits and with disease outcomes. Upper part; association estimates represent the beta coefficient (and its 95% confidence interval) in units of outcome trait for carriers of cAMP biased gain-of-function variants in *MC4R* compared to non-carriers (reference category). Lower part; Association estimates represent the odds ratio (and its 95% confidence interval) for carriers of cAMP biased gain-of-function variants in *MC4R* compared to non-carriers (reference category). Analyses were performed in European ancestry participants of UK Biobank. GoF, gain-of-function; n, number of participants; IQR, interquartile range; CI, confidence interval; OR, odds ratio; cAMP, cyclic adenosine monophosphate; BMI, body mass index; mmHg, millimeters of mercury; bpm, beats per minute.
