# Supplementary material for: Human Gain-of-Function MC4R Variants Show Signaling Bias and Protect against Obesity
Source: Cell. 2019 Apr 18;177(3):597–607.e9. doi: 10.1016/j.cell.2019.03.044 (PMC6476272; doi:10.1016/j.cell.2019.03.044)
Supplement: Table S7. Association of the Gain-of-Function Variant V103I MC4R with Continuous Traits and Disease Outcomes Related to Figures 3 and 4 [file mmc7.pdf]

**Table S7.** Association of the gain-of-function variant V103I MC4R with continuous traits and disease outcomes. Related to Figure 4.

| Outcome, unit | n | | Beta (95% CI) per copy of the variant (V103I) allele | *P* value |
|---|---|---|---|---|
| BMI, kg/m$^2$ | 450,708 | | -0.52 (-0.58, -0.45) | 1 x $10^{-54}$ |
| Systolic blood pressure, mmHg | 451,439 | | -0.02 (-0.28, 0.23) | 0.86 |
| Diastolic blood pressure, mmHg | 451,452 | | -0.21 (-0.36, -0.07) | 0.004 |
| Resting heart rate, bpm | 422,425 | | -0.28 (-0.45, -0.11) | 0.001 |
| **Outcome** | **Cases** | **Controls** | **OR (95% CI) per copy of the variant (V103I) allele** | ***P* value** |
| Obesity | 109,139 | 149,605 | 0.77 (0.75, 0.80) | 4 x $10^{-43}$ |
| Severe obesity | 8,496 | 149,605 | 0.69 (0.61, 0.77) | 9 x $10^{-10}$ |
| **Association with risk of type 2 diabetes** | | | | |
| **Study** | **Cases** | **Controls** | **OR (95% CI) per copy of the variant (V103I) allele** | ***P* value** |
| DIAGRAM | 34,840 | 114,981 | 0.88 (0.80, 0.96) | 0.004 |
| InterAct-Exome | 5,121 | 7,269 | 1.12 (0.92, 1.36) | 0.92 |
| InterAct-GWAS | 4,187 | 4,254 | 0.81 (0.63, 1.03) | 0.09 |
| UK Biobank | 24,758 | 424,575 | 0.86 (0.80, 0.92) | 1 x $10^{-05}$ |
| Meta-analysis ($P_{heterogeneity}$=0.08) | 68,906 | 551,079 | 0.88 (0.84, 0.93) | 7 x $10^{-07}$ |
| **Association with risk of coronary artery disease** | | | | |
| **Study** | **Cases** | **Controls** | **OR (95% CI) per copy of the variant (V103I) allele** | ***P* value** |
| CARDIoGRAMplusC4D | 60,801 | 123,504 | 0.94 (0.88, 1.01) | 0.09 |
| UK Biobank | 24,896 | 427,404 | 0.92 (0.86, 0.98) | 0.02 |
| Meta-analysis ($P_{heterogeneity}$=0.61) | 85,697 | 550,908 | 0.93 (0.89, 0.98) | 0.003 |

Upper part; Associations with continuous traits and risk of obesity and severe obesity. Analyses were performed in European ancestry participants of UK Biobank. Lower part; Association with risk of type 2 diabetes and coronary artery disease in meta-analyses of genetic association studies. Association estimates were generated in each study separately and were combined using fixed-effect inverse-variance weighted meta-analysis. The type 2 diabetes association meta-analysis included 68,906 cases and 551,079 controls from the DIAGRAM, EPIC-InterAct and UK Biobank studies. Results from sub-sets of InterAct genotyped using the Illumina CoreExome (InterAct-Exome) and Illumina 660w quad (Interact-GWAS) arrays are plotted separately. The coronary artery disease association meta-analysis included 85,697 cases and 550,908 controls from the CARDIoGRAMplusC4D and UK Biobank studies. n, number of participants; OR, odds ratio; CI, confidence interval; BMI, body mass index; mmHg, millimeters of mercury; bpm, beats per minute.
