# Supplementary material for: Human Gain-of-Function MC4R Variants Show Signaling Bias and Protect against Obesity
Source: Cell. 2019 Apr 18;177(3):597–607.e9. doi: 10.1016/j.cell.2019.03.044 (PMC6476272; doi:10.1016/j.cell.2019.03.044)
Supplement: Table S8. Functional Characterization of V103I MC4R, Related to Figure 4 [file mmc8.pdf]

Table S8. Functional characterization of V103I MC4R. Related to Figure 4.

assay	agonist	MC4R variant	agonist potency (M)		maximal activity [%WT]		*P* value	n
			mean	SEM	mean	SEM		
cAMP production	αMSH	WT	2.2E-09	2.8E-10	100%	n/a	0.009	4
		V103I	1.9E-09	1.6E-10	196%	25.3%		4
	βMSH	WT	2.3E-09	2.8E-10	100%	n/a	<0.001	4
		V103I	2.3E-09	4.3E-10	183%	11.1%		4
	NDP-αMSH	WT	1.7E-10	3.4E-11	100%	n/a	<0.001	4
		V103I	1.9E-10	3.6E-11	165%	9.2%		4
β-arrestin 2 recruitment	αMSH	WT	2.1E-08	1.1E-08	100%	n/a	<0.001	5
		V103I	7.4E-09	2.0E-09	244%	24%		5
	βMSH	WT	3.2E-08	1.3E-08	100%	n/a	<0.001	3
		V103I	1.9E-08	4.6E-09	348%	7.5%		3
	NDP-αMSH	WT	2.5E-09	8.8E-10	100%	n/a	0.001	5
		V103I	1.5E-09	3.1E-10	268%	35%		5

Dose-response curves were used to determine the potency of NDP-αMSH on V103I MC4R compared with wild-type. Relative agonist activity was estimated through normalization of the dose response curves to maximal NDP-αMSH-induced stimulation of wild-type receptor (100%) and basal activity in mock-transfected cells (0%). n/a, not applicable; n, number of experiments.
